# Supplementary material for: Evaluation of culture- and PCR-based methods for detecting Burkholderia pseudomallei in soil samples in Thailand
Source: PLoS Negl Trop Dis. 2026 Jan 2;20(1):e0013840. doi: 10.1371/journal.pntd.0013840 (PMC12758721; doi:10.1371/journal.pntd.0013840)
Supplement: S1 Table — The colony forming unit (CFU/ml) of eleven B. pseudomallei was measured at 3 and 7 days of incubation at 37 °C. The experiments were performed in triplicate in three independent assays. (DOCX) [file pntd.0013840.s001.docx]

**S1 Table**. **Colony count of eleven *B. pseudomallei* strains on Ashdown and ACER agar.** The colony forming unit (CFU/ml) of eleven *B. pseudomallei* was measured at 3 and 7 days of incubation at 37ºC. The experiments were performed in triplicate in three independent assays.

**First experiment (Day 3)**

| **Bacterial strains** | **Types of media** | **Colony count (CFU)** | | | **Average (CFU)** | **CFU/ml** |
| --- | --- | --- | --- | --- | --- | --- |
|  |  | **Replicate 1** | **Replicate 2** | **Replicate 3** |  |  |
| 30-191-S08 | Ashdown agar | 23 | 36 | 49 | 36 | 720 |
|  | ACER agar | 48 | 38 | 43 | 43 | 860 |
| 30-191-S17 | Ashdown agar | 45 | 45 | 37 | 42.33 | 846.67 |
|  | ACER agar | 54 | 60 | 54 | 56 | 1120 |
| 30-198-S23 | Ashdown agar | 45 | 47 | 49 | 47 | 940 |
|  | ACER agar | 40 | 38 | 58 | 45.33 | 906.67 |
| 30-194-S03 | Ashdown agar | 79 | 121 | 149 | 116.33 | 2326.67 |
|  | ACER agar | 179 | 158 | 116 | 151 | 3020 |
| 30-198-S28 | Ashdown agar | 105 | 118 | 121 | 114.67 | 2293.33 |
|  | ACER agar | 103 | 117 | 87 | 102.33 | 2046.67 |
| 30-194-S14 | Ashdown agar | 116 | 101 | 115 | 110.67 | 2213.33 |
|  | ACER agar | 108 | 144 | 139 | 130.33 | 2606.67 |
| 30-191-S10 | Ashdown agar | 124 | 116 | 139 | 126.33 | 2526.67 |
|  | ACER agar | 127 | 113 | 70 | 103.33 | 2066.67 |
| 30-191-S16 | Ashdown agar | 123 | 104 | 117 | 114.67 | 2293.33 |
|  | ACER agar | 111 | 123 | 127 | 120.33 | 2406.67 |
| 30-194-S04 | Ashdown agar | 115 | 93 | 107 | 105 | 2100 |
|  | ACER agar | 103 | 73 | 107 | 94.33 | 1886.67 |
| 30-198-S22 | Ashdown agar | 15 | 17 | 40 | 24 | 480 |
|  | ACER agar | 39 | 29 | 31 | 33 | 660 |
| K96243 | Ashdown agar | 57 | 65 | 66 | 62.67 | 1253.33 |
|  | ACER agar | 87 | 66 | 78 | 77 | 1540 |

**Second experiment (Day 3)**

| **Bacterial strains** | **Types of media** | **Colony count (CFU)** | | | **Average colony** | **CFU/ml** |
| --- | --- | --- | --- | --- | --- | --- |
|  |  | **Replicate 1** | **Replicate 2** | **Replicate 3** |  |  |
| 30-191-S08 | Ashdown agar | 152 | 135 | 133 | 140 | 2800 |
|  | ACER agar | 95 | 141 | 110 | 115.33 | 2306.67 |
| 30-191-S17 | Ashdown agar | 133 | 146 | 110 | 129.67 | 2593.33 |
|  | ACER agar | 166 | 156 | 184 | 168.67 | 3373.33 |
| 30-198-S23 | Ashdown agar | 63 | 47 | 72 | 60.67 | 1213.33 |
|  | ACER agar | 38 | 62 | 46 | 48.67 | 973.33 |
| 30-194-S03 | Ashdown agar | 83 | 101 | 91 | 91.67 | 1833.33 |
|  | ACER agar | 95 | 109 | 68 | 90.67 | 1813.33 |
| 30-198-S28 | Ashdown agar | 105 | 118 | 121 | 114.667 | 2293.33 |
|  | ACER agar | 103 | 117 | 87 | 102.33 | 2046.67 |
| 30-194-S14 | Ashdown agar | 116 | 101 | 115 | 110.67 | 2213.33 |
|  | ACER agar | 108 | 144 | 139 | 130.33 | 2606.67 |
| 30-191-S10 | Ashdown agar | 124 | 116 | 139 | 126.33 | 2526.67 |
|  | ACER agar | 127 | 113 | 70 | 103.33 | 2066.67 |
| 30-191-S16 | Ashdown agar | 77 | 84 | 65 | 75.33 | 1506.67 |
|  | ACER agar | 67 | 56 | 94 | 72.33 | 1446.67 |
| 30-194-S04 | Ashdown agar | 115 | 93 | 107 | 105 | 2100 |
|  | ACER agar | 103 | 73 | 107 | 94.33 | 1886.67 |
| 30-198-S22 | Ashdown agar | 81 | 74 | 86 | 80.33 | 1606.67 |
|  | ACER agar | 58 | 49 | 60 | 55.67 | 1113.33 |
| K96243 | Ashdown agar | 115 | 96 | 110 | 107 | 2140 |
|  | ACER agar | 130 | 121 | 80 | 110.33 | 2206.67 |

**Third experiment (Day 3)**

| **Bacterial strains** | **Types of media** | **Colony count (CFU)** | | | **Average (CFU)** | **CFU/ml** |
| --- | --- | --- | --- | --- | --- | --- |
|  |  | **Replicate 1** | **Replicate 2** | **Replicate 3** |  |  |
| 30-191-S08 | Ashdown agar | 115 | 125 | 122 | 120.67 | 2413.33 |
|  | ACER agar | 123 | 148 | 138 | 136.33 | 2726.67 |
| 30-191-S17 | Ashdown agar | 139 | 161 | 152 | 150.67 | 3013.33 |
|  | ACER agar | 169 | 167 | 146 | 160.67 | 3213.33 |
| 30-198-S23 | Ashdown agar | 127 | 106 | 122 | 118.33 | 2366.67 |
|  | ACER agar | 129 | 141 | 135 | 135 | 2700 |
| 30-194-S03 | Ashdown agar | 137 | 187 | 163 | 162.33 | 3246.67 |
|  | ACER agar | 179 | 174 | 124 | 159 | 3180 |
| 30-198-S28 | Ashdown agar | 133 | 138 | 112 | 127.67 | 2553.33 |
|  | ACER agar | 137 | 161 | 150 | 149.33 | 2986.67 |
| 30-194-S14 | Ashdown agar | 148 | 121 | 146 | 138.33 | 2766.67 |
|  | ACER agar | 133 | 170 | 154 | 152.33 | 3046.67 |
| 30-191-S10 | Ashdown agar | 123 | 123 | 125 | 123.67 | 2473.33 |
|  | ACER agar | 124 | 78 | 117 | 106.33 | 2126.67 |
| 30-191-S16 | Ashdown agar | 151 | 145 | 148 | 148 | 2960 |
|  | ACER agar | 181 | 152 | 156 | 163 | 3260 |
| 30-194-S04 | Ashdown agar | 115 | 109 | 128 | 117.33 | 2346.67 |
|  | ACER agar | 132 | 170 | 187 | 163 | 3260 |
| 30-198-S22 | Ashdown agar | 120 | 146 | 119 | 128.33 | 2566.67 |
|  | ACER agar | 104 | 83 | 67 | 84.67 | 1693.33 |
| K96243 | Ashdown agar | 120 | 125 | 122 | 122.33 | 2446.67 |
|  | ACER agar | 130 | 145 | 138 | 137.67 | 2753.33 |

**First experiment (Day 7)**

| **Bacterial strains** | **Types of media** | **Colony count (CFU)** | | | **Average (CFU)** | **CFU/ml** |
| --- | --- | --- | --- | --- | --- | --- |
|  |  | **Replicate 1** | **Replicate 2** | **Replicate 3** |  |  |
| 30-191-S08 | Ashdown agar | 23 | 36 | 49 | 36 | 720 |
|  | ACER agar | 48 | 38 | 43 | 43 | 860 |
| 30-191-S17 | Ashdown agar | 45 | 45 | 37 | 42.33 | 846.67 |
|  | ACER agar | 54 | 60 | 54 | 56 | 1120 |
| 30-198-S23 | Ashdown agar | 45 | 47 | 49 | 47 | 940 |
|  | ACER agar | 40 | 38 | 58 | 45.33 | 906.67 |
| 30-194-S03 | Ashdown agar | 79 | 121 | 149 | 116.33 | 2326.67 |
|  | ACER agar | 179 | 158 | 116 | 151 | 3020 |
| 30-198-S28 | Ashdown agar | 105 | 118 | 121 | 114.67 | 2293.33 |
|  | ACER agar | 103 | 117 | 87 | 102.33 | 2046.67 |
| 30-194-S14 | Ashdown agar | 116 | 101 | 115 | 110.67 | 2213.33 |
|  | ACER agar | 108 | 144 | 139 | 130.33 | 2606.67 |
| 30-191-S10 | Ashdown agar | 124 | 116 | 139 | 126.33 | 2526.67 |
|  | ACER agar | 127 | 113 | 70 | 103.33 | 2066.67 |
| 30-191-S16 | Ashdown agar | 123 | 104 | 117 | 114.67 | 2293.33 |
|  | ACER agar | 111 | 123 | 127 | 120.33 | 2406.67 |
| 30-194-S04 | Ashdown agar | 115 | 93 | 107 | 105 | 2100 |
|  | ACER agar | 103 | 73 | 107 | 94.33 | 1886.67 |
| 30-198-S22 | Ashdown agar | 15 | 17 | 40 | 24 | 480 |
|  | ACER agar | 39 | 29 | 31 | 33 | 660 |
| K96243 | Ashdown agar | 57 | 65 | 66 | 62.67 | 1253.33 |
|  | ACER agar | 87 | 66 | 78 | 77 | 1540 |

**Second experiment (Day 7)**

| **Bacterial strains** | **Types of media** | **Colony count (CFU)** | | | **Average (CFU)** | **CFU/ml** |
| --- | --- | --- | --- | --- | --- | --- |
|  |  | **Replicate 1** | **Replicate 2** | **Replicate 3** |  |  |
| 30-191-S08 | Ashdown agar | 152 | 135 | 133 | 140 | 2800 |
|  | ACER agar | 95 | 141 | 110 | 115.33 | 2306.67 |
| 30-191-S17 | Ashdown agar | 133 | 146 | 110 | 129.67 | 2593.33 |
|  | ACER agar | 166 | 156 | 184 | 168.67 | 3373.33 |
| 30-198-S23 | Ashdown agar | 63 | 47 | 72 | 60.67 | 1213.33 |
|  | ACER agar | 38 | 62 | 46 | 48.67 | 973.33 |
| 30-194-S03 | Ashdown agar | 83 | 101 | 91 | 91.67 | 1833.33 |
|  | ACER agar | 95 | 109 | 68 | 90.67 | 1813.33 |
| 30-198-S28 | Ashdown agar | 105 | 118 | 121 | 114.667 | 2293.33 |
|  | ACER agar | 103 | 117 | 87 | 102.33 | 2046.67 |
| 30-194-S14 | Ashdown agar | 116 | 101 | 115 | 110.67 | 2213.33 |
|  | ACER agar | 108 | 144 | 139 | 130.33 | 2606.67 |
| 30-191-S10 | Ashdown agar | 124 | 116 | 139 | 126.33 | 2526.67 |
|  | ACER agar | 127 | 113 | 70 | 103.33 | 2066.67 |
| 30-191-S16 | Ashdown agar | 77 | 84 | 65 | 75.33 | 1506.67 |
|  | ACER agar | 67 | 56 | 94 | 72.33 | 1446.67 |
| 30-194-S04 | Ashdown agar | 115 | 93 | 107 | 105 | 2100 |
|  | ACER agar | 103 | 73 | 107 | 94.33 | 1886.67 |
| 30-198-S22 | Ashdown agar | 81 | 74 | 86 | 80.33 | 1606.67 |
|  | ACER agar | 58 | 49 | 60 | 55.67 | 1113.33 |
| K96243 | Ashdown agar | 115 | 96 | 110 | 107 | 2140 |
|  | ACER agar | 130 | 121 | 80 | 110.33 | 2206.67 |

**Third experiment (Day 7)**

| **Bacterial strains** | **Types of media** | **Colony count (CFU)** | | | **Average (CFU)** | **CFU/ml** |
| --- | --- | --- | --- | --- | --- | --- |
|  |  | **Replicate 1** | **Replicate 2** | **Replicate 3** |  |  |
| 30-191-S08 | Ashdown agar | 115 | 125 | 122 | 120.67 | 2413.33 |
|  | ACER agar | 123 | 148 | 138 | 136.33 | 2726.67 |
| 30-191-S17 | Ashdown agar | 139 | 161 | 152 | 150.67 | 3013.33 |
|  | ACER agar | 169 | 167 | 146 | 160.67 | 3213.33 |
| 30-198-S23 | Ashdown agar | 127 | 106 | 122 | 118.33 | 2366.67 |
|  | ACER agar | 129 | 141 | 135 | 135 | 2700 |
| 30-194-S03 | Ashdown agar | 137 | 187 | 163 | 162.33 | 3246.67 |
|  | ACER agar | 179 | 174 | 124 | 159 | 3180 |
| 30-198-S28 | Ashdown agar | 133 | 138 | 112 | 127.67 | 2553.33 |
|  | ACER agar | 137 | 161 | 150 | 149.33 | 2986.67 |
| 30-194-S14 | Ashdown agar | 148 | 121 | 146 | 138.33 | 2766.67 |
|  | ACER agar | 133 | 170 | 154 | 152.33 | 3046.67 |
| 30-191-S10 | Ashdown agar | 123 | 123 | 125 | 123.67 | 2473.33 |
|  | ACER agar | 124 | 78 | 117 | 106.33 | 2126.67 |
| 30-191-S16 | Ashdown agar | 151 | 145 | 148 | 148 | 2960 |
|  | ACER agar | 181 | 152 | 156 | 163 | 3260 |
| 30-194-S04 | Ashdown agar | 115 | 109 | 128 | 117.33 | 2346.67 |
|  | ACER agar | 132 | 170 | 187 | 163 | 3260 |
| 30-198-S22 | Ashdown agar | 120 | 146 | 119 | 128.33 | 2566.67 |
|  | ACER agar | 104 | 83 | 67 | 84.67 | 1693.33 |
| K96243 | Ashdown agar | 120 | 125 | 122 | 122.33 | 2446.67 |
|  | ACER agar | 130 | 145 | 138 | 137.67 | 2753.33 |
