# Supplementary material for: Evaluation of culture- and PCR-based methods for detecting Burkholderia pseudomallei in soil samples in Thailand
Source: PLoS Negl Trop Dis. 2026 Jan 2;20(1):e0013840. doi: 10.1371/journal.pntd.0013840 (PMC12758721; doi:10.1371/journal.pntd.0013840)
Supplement: S1 Fig — The growth of B. pseudomallei was measured at 3 and 7 days of incubation at 37 °C in air. The experiments were performed in triplicate in three independent assays. (PDF) [file pntd.0013840.s002.pdf]

***B. pseudomallei* 30-191-S08**

Ashdown agar | **ACER agar**

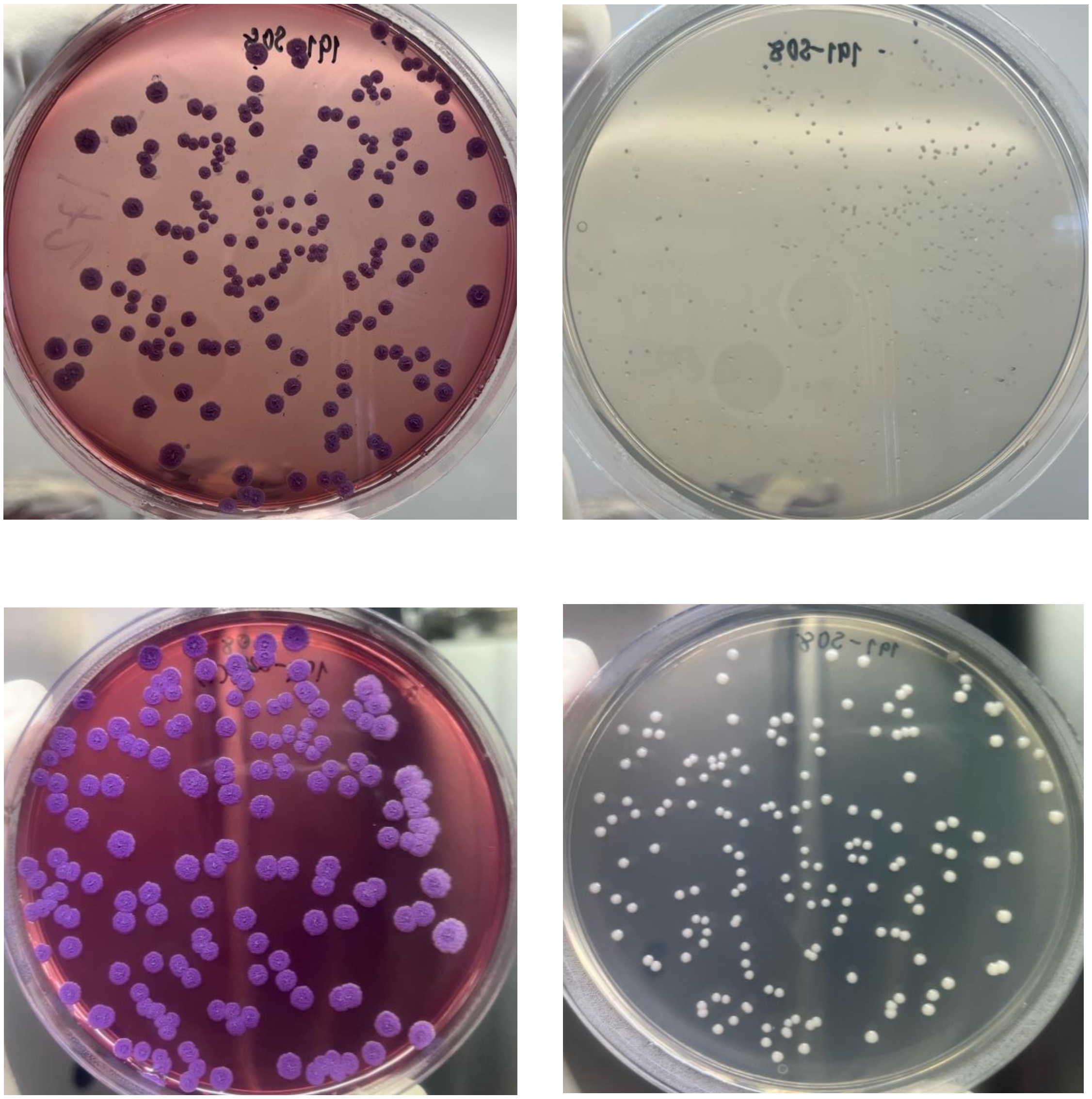

Day 3

Day 7

***B. pseudomallei* 30-191-S10**

Ashdown agar **ACER agar**

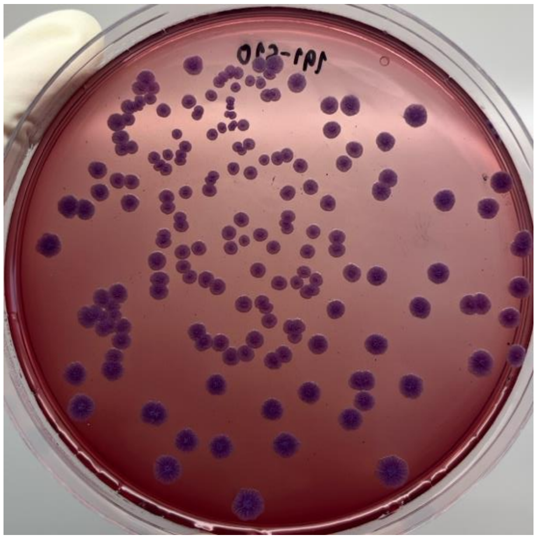

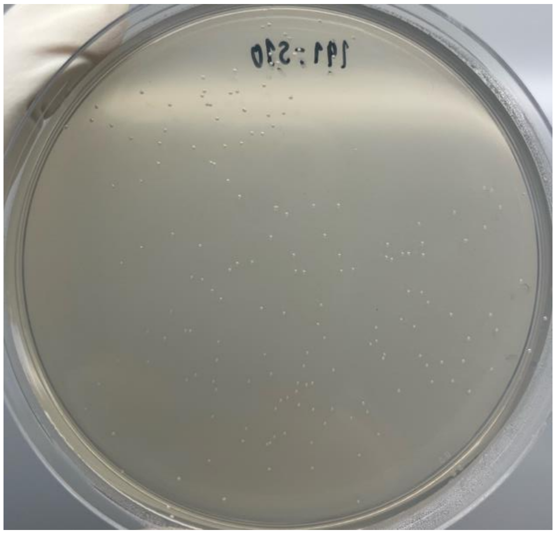

Day 3

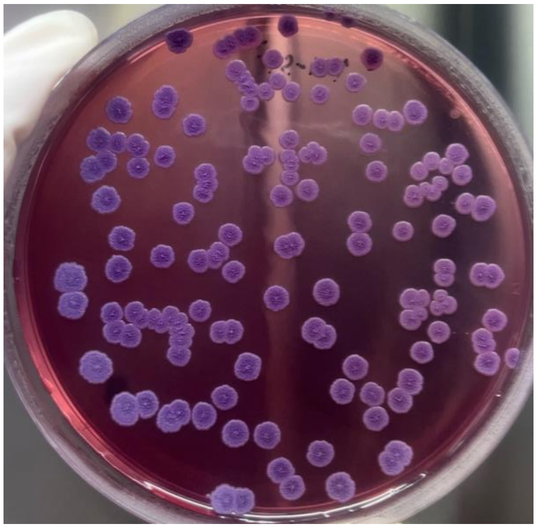

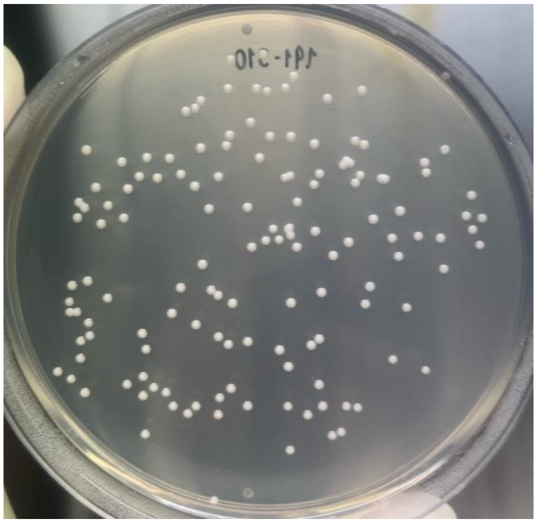

Day 7

***B. pseudomallei* 30-191-S16**

Ashdown agar	ACER agar	
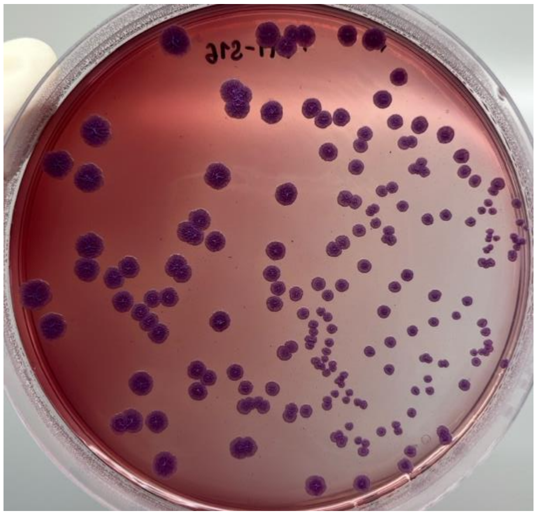	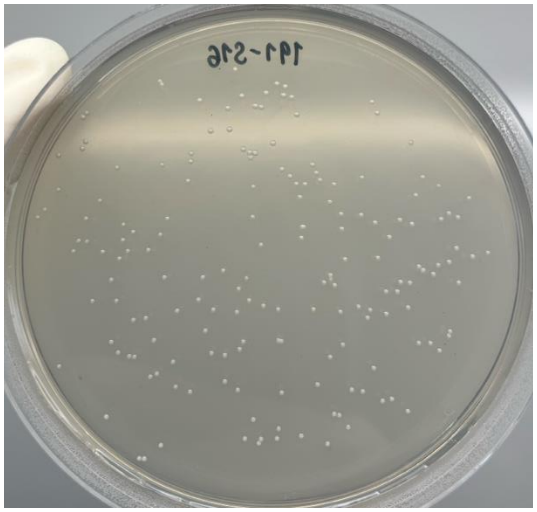	**Day 3**
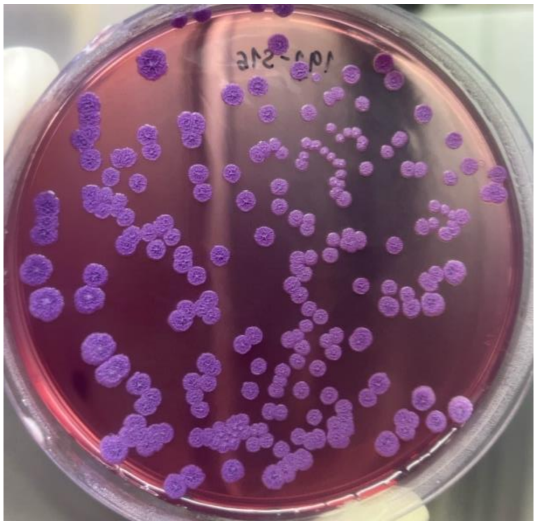	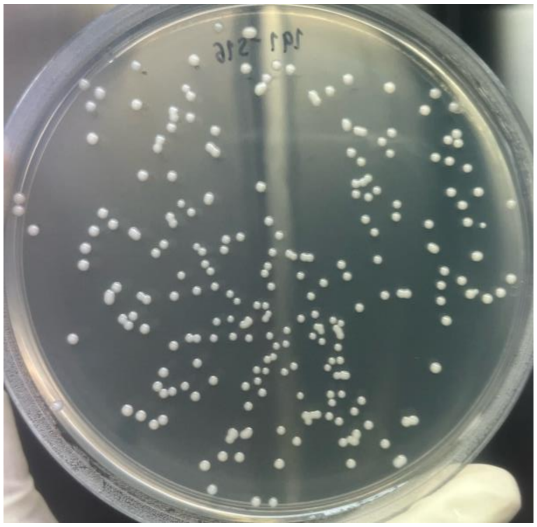	**Day 7**

***B. pseudomallei* 30-191-S17**

Ashdown agar **ACER agar**

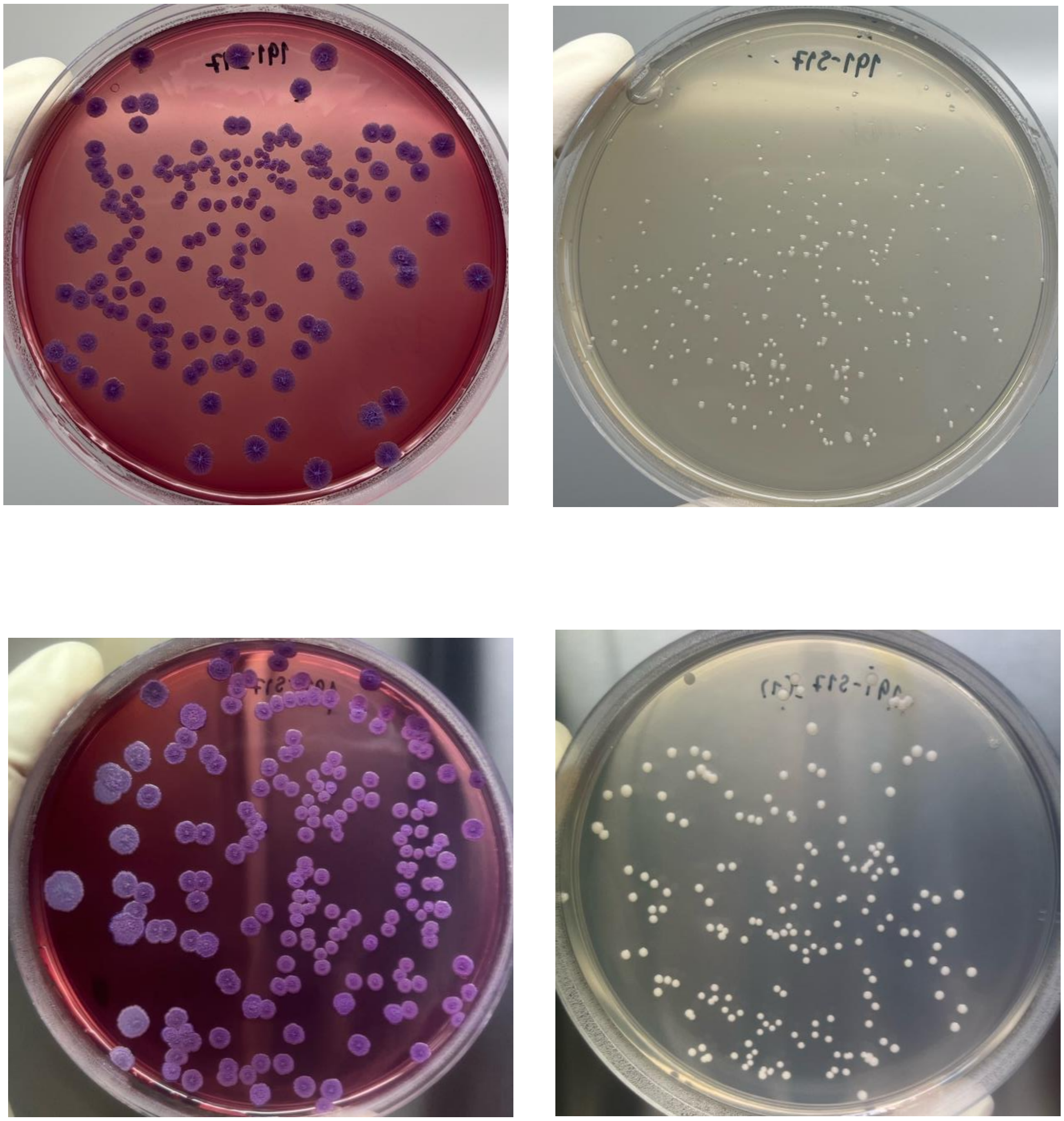

Day 3

Day 7

***B. pseudomallei* 30-194-S03**

Ashdown agar | **ACER agar**

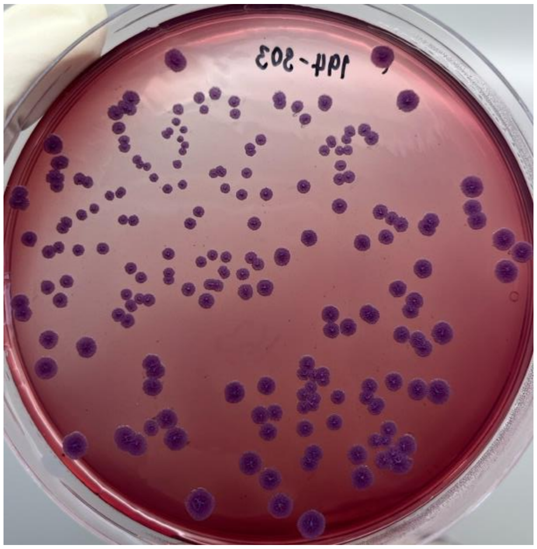

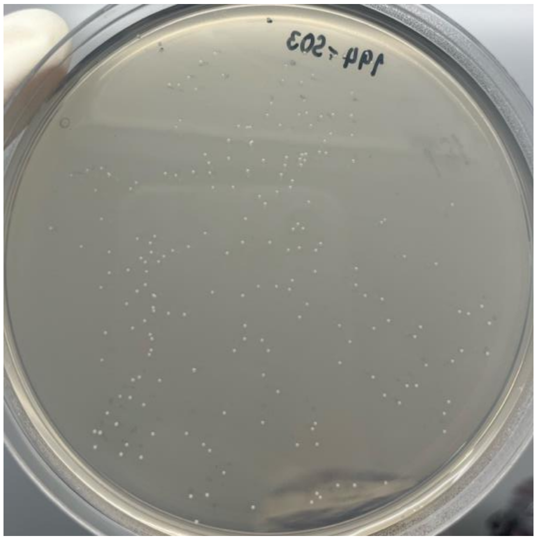

Day 3

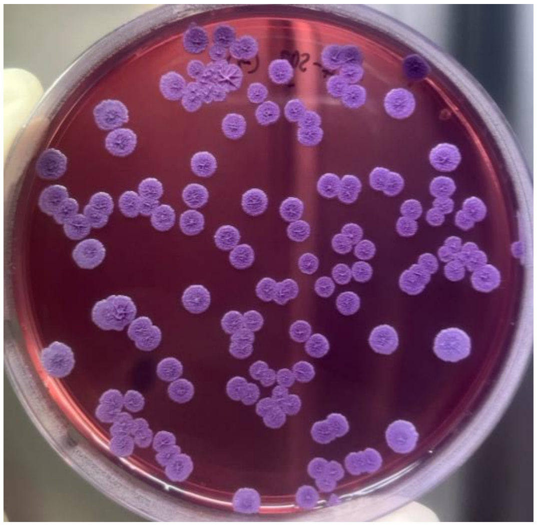

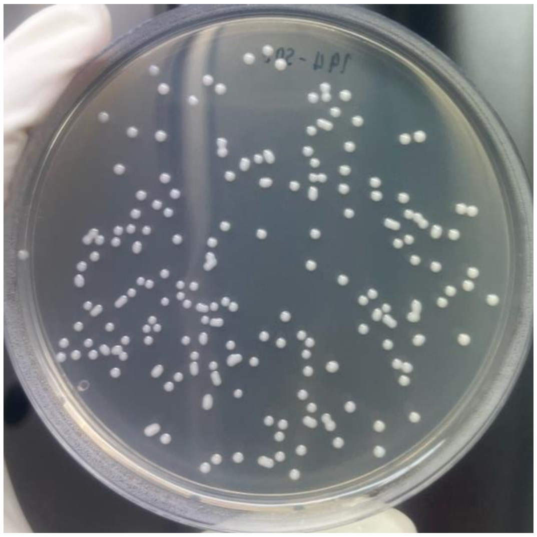

Day 7

B. pseudomallei **30-194-S04**

Ashdown agar | **ACER agar**

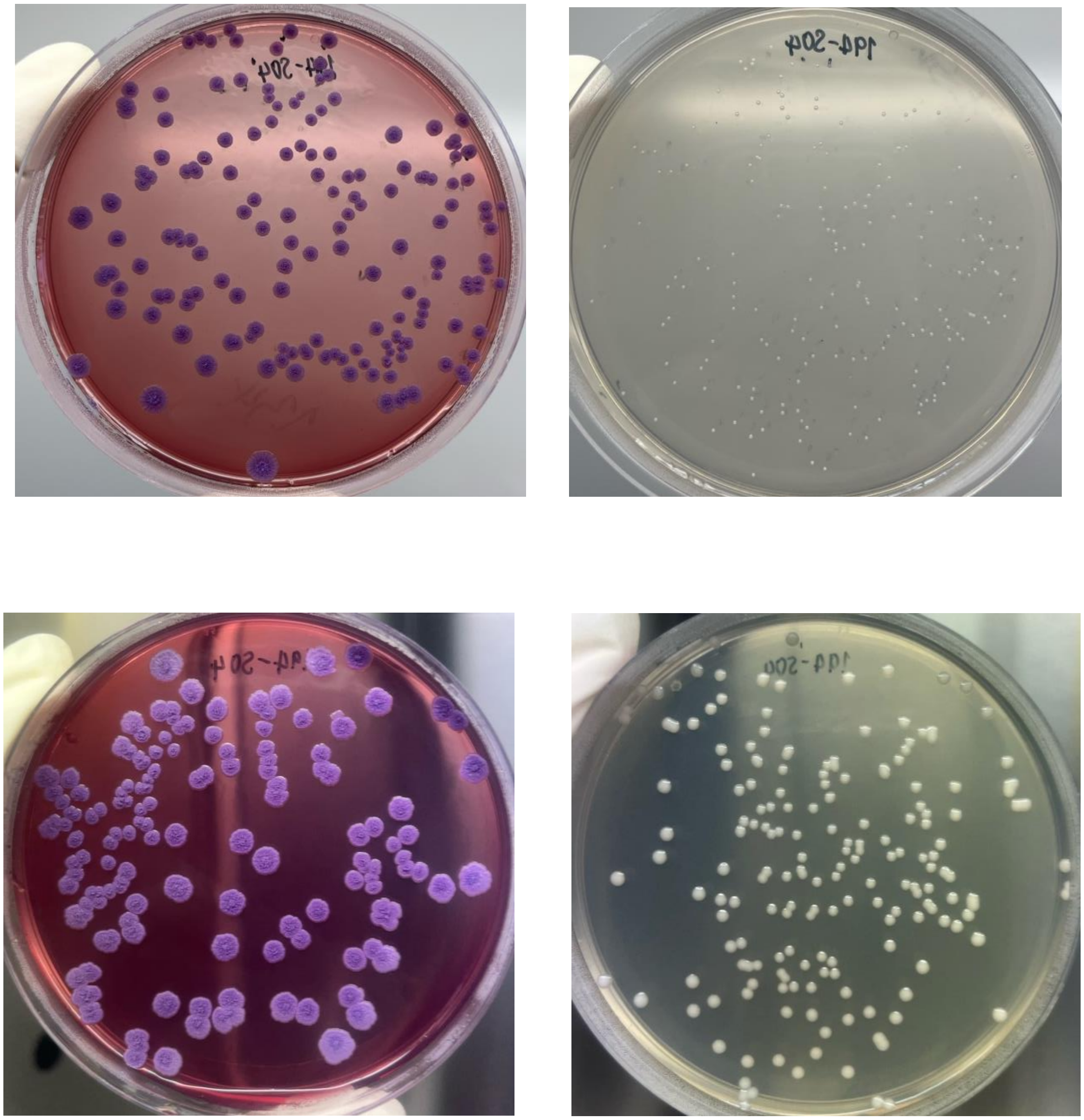

Day 3

Day 7

B. pseudomallei 30-194-S14

Ashdown agar | **ACER agar**

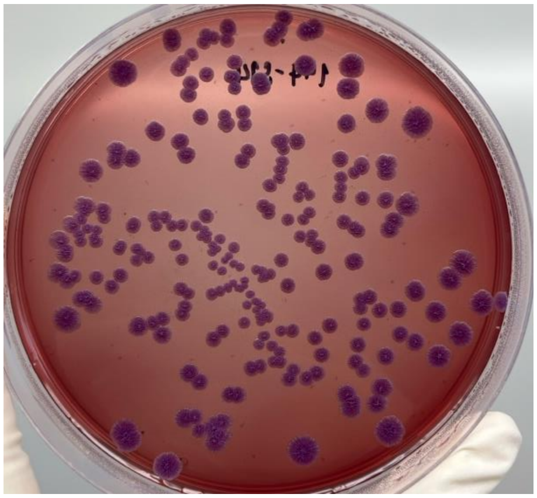

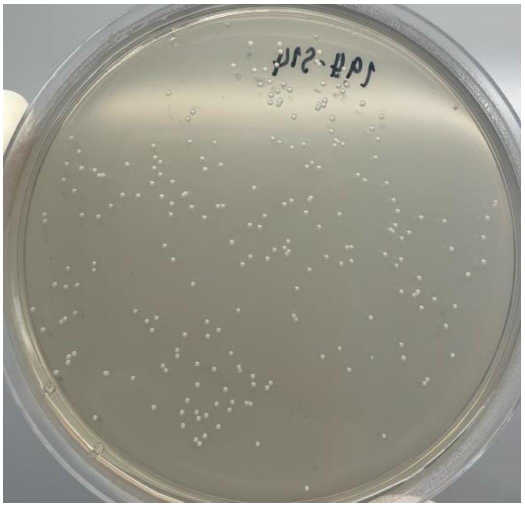

Day 3

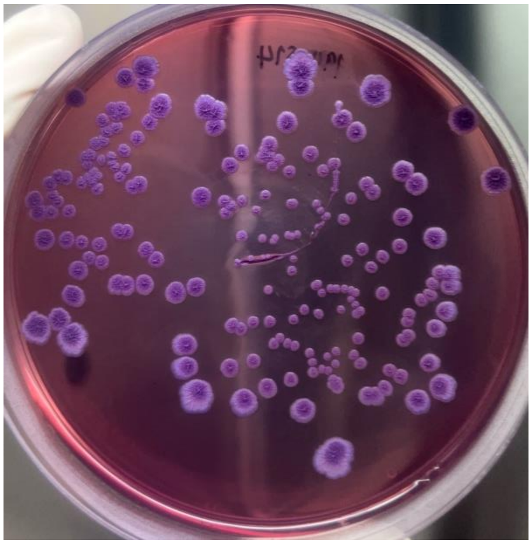

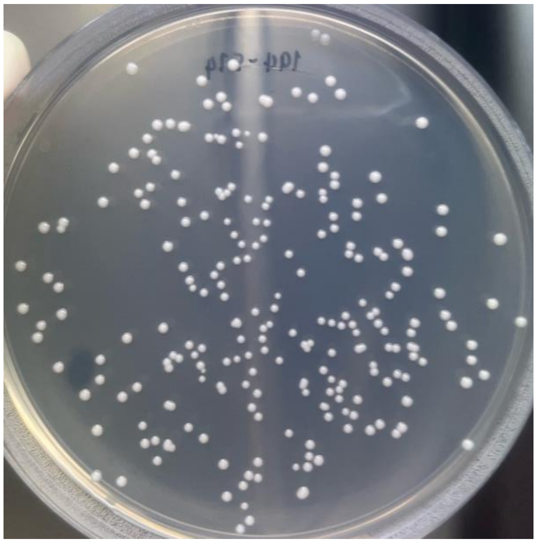

Day 7

***B. pseudomallei* 30-198-S22**

Ashdown agar **ACER agar**

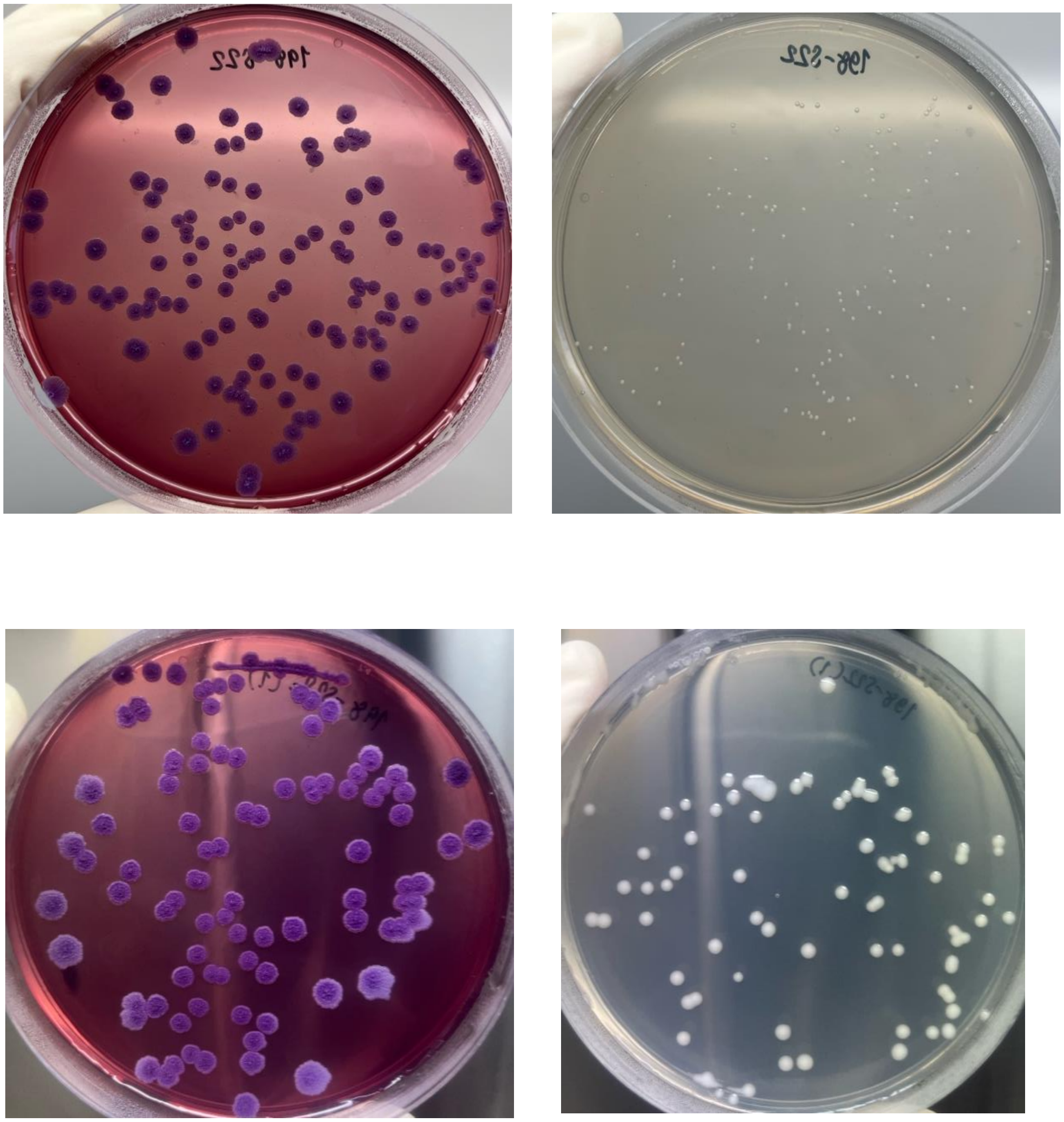

Day 3

Day 7

***B. pseudomallei* 30-198-S23**

Ashdown agar	ACER agar	
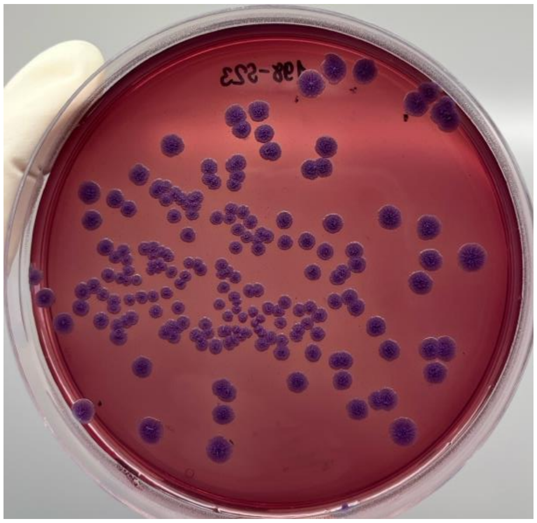	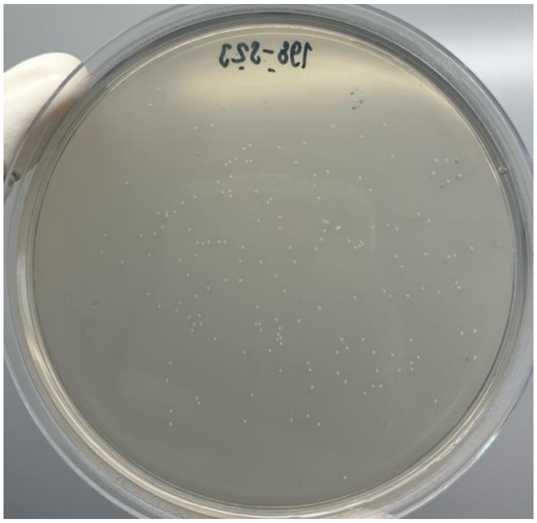	**Day 3**
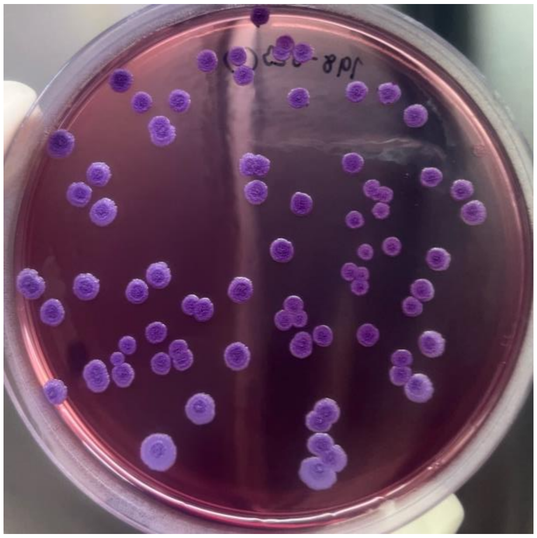	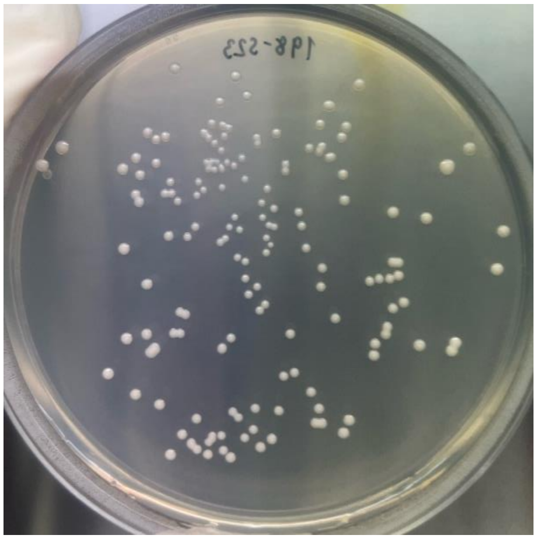	**Day 7**

***B. pseudomallei* 30-198- S28**

Ashdown agar | **ACER agar**

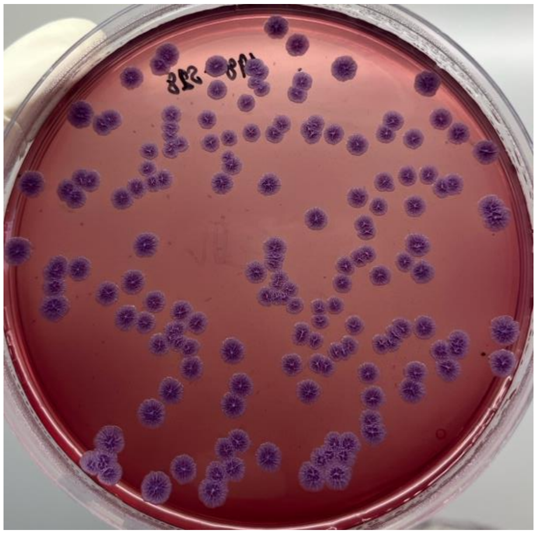

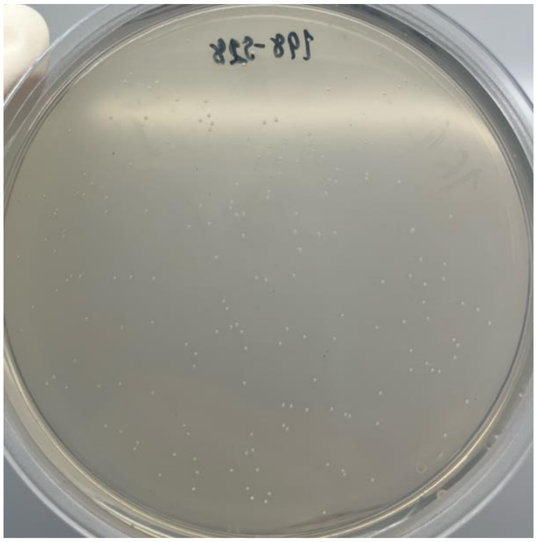

Day 3

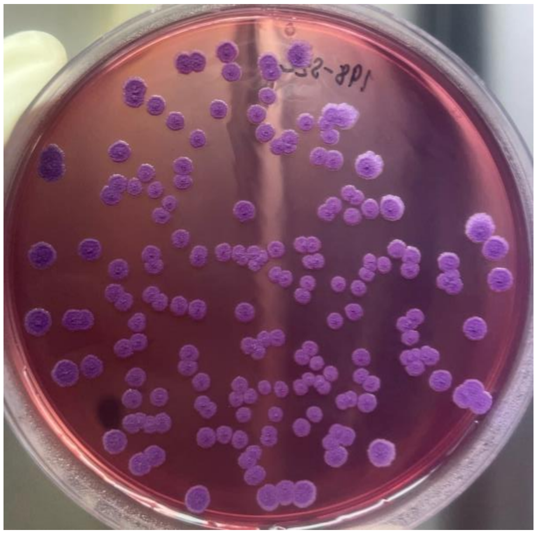

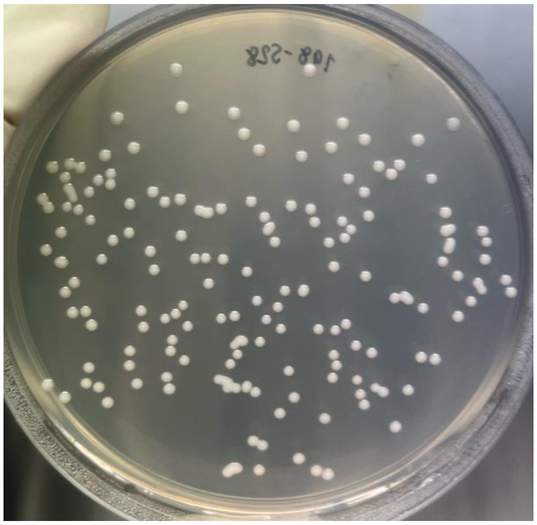

Day 7

***B. pseudomallei* K96243**

Ashdown agar **ACER agar**

Day 3

Day 7

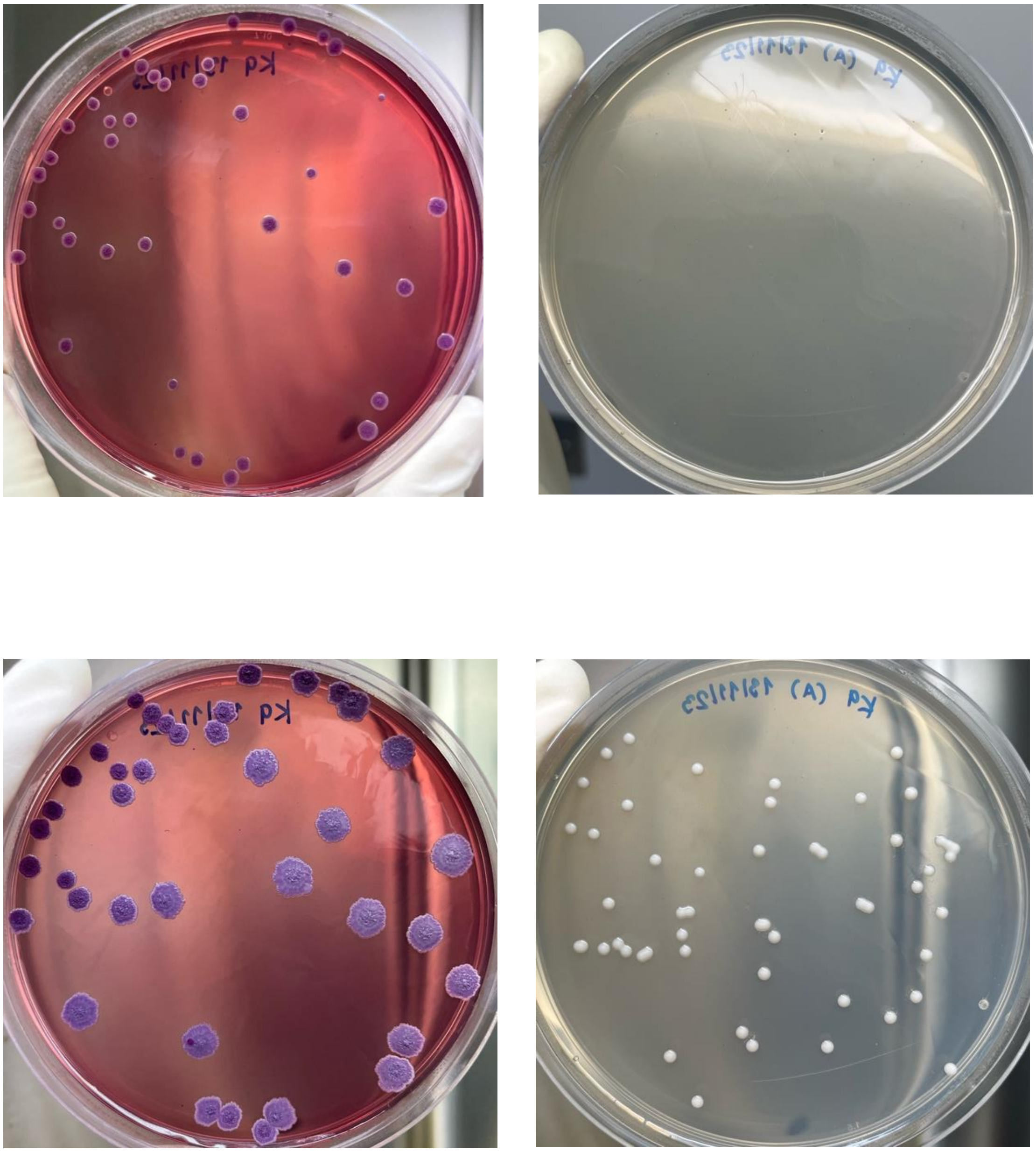

S1 Fig. Colony morphology of eleven *B. pseudomallei* strains on Ashdown and ACER agar. The growth of *B. pseudomallei* was measured after 3 days and 7 days of incubation at 37ºC in air. The experiments were performed in triplicate in three independent assays.
