# Supplementary material for: Evaluation of culture- and PCR-based methods for detecting Burkholderia pseudomallei in soil samples in Thailand
Source: PLoS Negl Trop Dis. 2026 Jan 2;20(1):e0013840. doi: 10.1371/journal.pntd.0013840 (PMC12758721; doi:10.1371/journal.pntd.0013840)
Supplement: S2 Table — The log colony forming unit (log CFU/ml) of eleven B. pseudomallei was measured at 2, 5 and 9 days of incubation at 37 °C. The experiments were performed in triplicate in three independent assays. (DOCX) [file pntd.0013840.s003.docx]

**S2 Table. Colony count of eleven B. pseudomallei strains on TBSS-C50, TBSS-C50 based erythritol and ACER enrichment broth.** The log colony forming unit (log CFU/ml) of eleven *B. pseudomallei* was measured at 2, 5 and 9 days of incubation at 37ºC. The experiments were performed in triplicate in three independent assays.

**Day 2**

| **Bacterial strains** | **Types of media** | **Colony count (CFU)** | | | **Average (CFU)** | **CFU/ml** | **LogCFU/ml** |
| --- | --- | --- | --- | --- | --- | --- | --- |
|  |  | **Replicate 1** | **Replicate 2** | **Replicate 3** |  |  |  |
| **30-191-S08** | TBSS-C50 | 8 | 7 | 4 | 6.33 | 63333333 | 7.80 |
|  | EM | 2 | 2 | 3 | 2.33 | 23333333 | 7.37 |
|  | ACER | 2 | 5 | 9 | 5.33 | 53333333 | 7.73 |
| **30-191-S17** | TBSS-C50 | 8 | 7 | 5 | 6.67 | 66666667 | 7.82 |
|  | EM | 5 | 2 | 0 | 2.33 | 23333333 | 7.37 |
|  | ACER | 10 | 11 | 12 | 11.00 | 110000000 | 8.04 |
| **30-198-S23** | TBSS-C50 | 7 | 4 | 6 | 5.67 | 56666667 | 7.75 |
|  | EM | 3 | 3 | 3 | 3.00 | 30000000 | 7.48 |
|  | ACER | 7 | 9 | 15 | 10.33 | 103333333 | 8.01 |
| **30-194-S03** | TBSS-C50 | 6 | 6 | 5 | 5.67 | 56666667 | 7.75 |
|  | EM | 6 | 4 | 2 | 4.00 | 40000000 | 7.60 |
|  | ACER | 7 | 8 | 10 | 8.33 | 83333333 | 7.92 |
| **30-198-S28** | TBSS-C50 | 13 | 10 | 10 | 11.00 | 110000000 | 8.04 |
|  | EM | 3 | 5 | 1 | 3.00 | 30000000 | 7.48 |
|  | ACER | 10 | 9 | 11 | 10.00 | 100000000 | 8.00 |
| **30-194-S14** | TBSS-C50 | 12 | 9 | 8 | 9.67 | 96666667 | 7.99 |
|  | EM | 2 | 0 | 0 | 0.67 | 6666667 | 6.82 |
|  | ACER | 12 | 9 | 8 | 9.67 | 96666667 | 7.99 |
| **30-191-S10** | TBSS-C50 | 5 | 6 | 7 | 6.00 | 60000000 | 7.78 |
|  | EM | 5 | 3 | 1 | 3.00 | 30000000 | 7.48 |
|  | ACER | 11 | 13 | 14 | 12.67 | 126666667 | 8.10 |
| **30-191-S16** | TBSS-C50 | 6 | 11 | 9 | 8.67 | 86666667 | 7.94 |
|  | EM | 1 | 3 | 2 | 2.00 | 20000000 | 7.30 |
|  | ACER | 8 | 9 | 10 | 9.00 | 90000000 | 7.95 |
| **30-194-S04** | TBSS-C50 | 8 | 8 | 4 | 6.67 | 66666667 | 7.82 |
|  | EM | 5 | 1 | 0 | 2.00 | 20000000 | 7.30 |
|  | ACER | 10 | 13 | 8 | 10.33 | 103333333 | 8.01 |
| **30-198-S22** | TBSS-C50 | 7 | 7 | 3 | 5.67 | 56666667 | 7.75 |
|  | EM | 5 | 2 | 3 | 3.33 | 33333333 | 7.52 |
|  | ACER | 7 | 10 | 9 | 8.67 | 86666667 | 7.94 |
| **K96243** | TBSS-C50 | 3 | 6 | 4 | 4.33 | 43333333 | 7.64 |
|  | EM | 2 | 0 | 0 | 0.67 | 6666667 | 6.82 |
|  | ACER | 5 | 7 | 7 | 6.33 | 63333333 | 7.80 |

**Day 5**

| **Bacterial strains** | **Types of media** | **Colony count (CFU)** | | | **Average (CFU)** | **CFU/ml** | **LogCFU/ml** |
| --- | --- | --- | --- | --- | --- | --- | --- |
|  |  | **Replicate 1** | **Replicate 2** | **Replicate 3** |  |  |  |
| **30-191-S08** | TBSS-C50 | 11 | 11 | 7 | 9.67 | 96666667 | 7.99 |
|  | EM | 4 | 5 | 6 | 5 | 50000000 | 7.70 |
|  | ACER | 11 | 11 | 16 | 12.67 | 126666667 | 8.10 |
| **30-191-S17** | TBSS-C50 | 7 | 4 | 6 | 5.67 | 56666667 | 7.75 |
|  | EM | 6 | 6 | 5 | 5.67 | 56666667 | 7.75 |
|  | ACER | 15 | 15 | 17 | 15.67 | 156666667 | 8.19 |
| **30-198-S23** | TBSS-C50 | 8 | 3 | 15 | 8.67 | 86666667 | 7.94 |
|  | EM | 14 | 13 | 8 | 11.67 | 116666667 | 8.07 |
|  | ACER | 14 | 12 | 12 | 12.67 | 126666667 | 8.10 |
| **30-194-S03** | TBSS-C50 | 9 | 20 | 15 | 14.67 | 146666667 | 8.17 |
|  | EM | 6 | 4 | 2 | 4.00 | 40000000 | 7.60 |
|  | ACER | 20 | 20 | 14 | 18.00 | 180000000 | 8.26 |
| **30-198-S28** | TBSS-C50 | 18 | 16 | 10 | 14.67 | 146666667 | 8.17 |
|  | EM | 8 | 7 | 2 | 5.67 | 56666667 | 7.75 |
|  | ACER | 2 | 4 | 4 | 3.33 | 33333333 | 7.52 |
| **30-194-S14** | TBSS-C50 | 12 | 13 | 11 | 12.00 | 120000000 | 8.08 |
|  | EM | 8 | 7 | 6 | 7.00 | 70000000 | 7.85 |
|  | ACER | 24 | 24 | 22 | 23.33 | 233333333 | 8.37 |
| **30-191-S10** | TBSS-C50 | 15 | 14 | 17 | 15.33 | 153333333 | 8.19 |
|  | EM | 4 | 3 | 9 | 5.33 | 53333333 | 7.73 |
|  | ACER | 14 | 17 | 18 | 16.33 | 163333333 | 8.21 |
| **30-191-S16** | TBSS-C50 | 13 | 15 | 17 | 15.00 | 150000000 | 8.18 |
|  | EM | 12 | 14 | 17 | 14.33 | 143333333 | 8.16 |
|  | ACER | 26 | 23 | 27 | 25.33 | 253333333 | 8.40 |
| **30-194-S04** | TBSS-C50 | 10 | 12 | 5 | 9.00 | 90000000 | 7.95 |
|  | EM | 7 | 4 | 11 | 7.33 | 73333333 | 7.87 |
|  | ACER | 20 | 24 | 19 | 21.00 | 210000000 | 8.32 |
| **30-198-S22** | TBSS-C50 | 14 | 14 | 19 | 15.67 | 156666667 | 8.19 |
|  | EM | 13 | 17 | 11 | 13.67 | 136666667 | 8.14 |
|  | ACER | 24 | 24 | 19 | 22.33 | 223333333 | 8.35 |
| **K96243** | TBSS-C50 | 9 | 8 | 14 | 10.33 | 103333333 | 8.01 |
|  | EM | 9 | 10 | 12 | 10.33 | 103333333 | 8.01 |
|  | ACER | 15 | 11 | 10 | 12.00 | 120000000 | 8.08 |

**Day 9**

| **Bacterial strains** | **Types of media** | **Colony count (CFU)** | | | **Average (CFU)** | **CFU/ml** | **LogCFU/ml** |
| --- | --- | --- | --- | --- | --- | --- | --- |
|  |  | **Replicate 1** | **Replicate 2** | **Replicate 3** |  |  |  |
| **30-191-S08** | TBSS-C50 | 2 | 1 | 5 | 2.67 | 26666667 | 7.43 |
|  | EM | 16 | 12 | 10 | 12.67 | 126666667 | 8.10 |
|  | ACER | 4 | 3 | 3 | 3.33 | 33333333 | 7.52 |
| **30-191-S17** | TBSS-C50 | 4 | 4 | 6 | 4.67 | 46666667 | 7.67 |
|  | EM | 9 | 10 | 13 | 10.67 | 106666667 | 8.03 |
|  | ACER | 18 | 16 | 14 | 16.00 | 160000000 | 8.20 |
| **30-198-S23** | TBSS-C50 | 12 | 13 | 14 | 13.00 | 130000000 | 8.11 |
|  | EM | 14 | 11 | 13 | 12.67 | 126666667 | 8.10 |
|  | ACER | 13 | 11 | 16 | 13.33 | 133333333 | 8.12 |
| **30-194-S03** | TBSS-C50 | 13 | 14 | 15 | 14.00 | 140000000 | 8.15 |
|  | EM | 10 | 10 | 5 | 8.33 | 83333333 | 7.92 |
|  | ACER | 16 | 13 | 21 | 16.67 | 166666667 | 8.22 |
| **30-198-S28** | TBSS-C50 | 14 | 16 | 9 | 13.00 | 130000000 | 8.11 |
|  | EM | 8 | 9 | 11 | 9.33 | 93333333 | 7.97 |
|  | ACER | 3 | 5 | 5 | 4.33 | 43333333 | 7.64 |
| **30-194-S14** | TBSS-C50 | 17 | 15 | 11 | 14.33 | 143333333 | 8.16 |
|  | EM | 11 | 11 | 12 | 11.33 | 113333333 | 8.05 |
|  | ACER | 20 | 27 | 17 | 21.33 | 213333333 | 8.33 |
| **30-191-S10** | TBSS-C50 | 22 | 18 | 17 | 19.00 | 190000000 | 8.28 |
|  | EM | 8 | 14 | 16 | 12.67 | 126666667 | 8.10 |
|  | ACER | 20 | 22 | 17 | 19.67 | 196666667 | 8.29 |
| **30-191-S16** | TBSS-C50 | 3 | 3 | 5 | 3.67 | 36666667 | 7.56 |
|  | EM | 11 | 13 | 13 | 12.33 | 123333333 | 8.09 |
|  | ACER | 2 | 8 | 1 | 3.67 | 36666667 | 7.56 |
| **30-194-S04** | TBSS-C50 | 12 | 16 | 18 | 15.33 | 153333333 | 8.19 |
|  | EM | 13 | 11 | 8 | 10.67 | 106666667 | 8.03 |
|  | ACER | 4 | 5 | 4 | 4.33 | 43333333 | 7.64 |
| **30-198-S22** | TBSS-C50 | 17 | 18 | 18 | 17.67 | 176666667 | 8.25 |
|  | EM | 15 | 17 | 14 | 15.33 | 153333333 | 8.19 |
|  | ACER | 14 | 16 | 18 | 16.00 | 160000000 | 8.20 |
| **K96243** | TBSS-C50 | 19 | 13 | 5 | 12.33 | 123333333 | 8.09 |
|  | EM | 11 | 12 | 9 | 10.67 | 106666667 | 8.03 |
|  | ACER | 8 | 10 | 10 | 9.33 | 93333333 | 7.97 |
