# Supplementary material for: Evaluation of culture- and PCR-based methods for detecting Burkholderia pseudomallei in soil samples in Thailand
Source: PLoS Negl Trop Dis. 2026 Jan 2;20(1):e0013840. doi: 10.1371/journal.pntd.0013840 (PMC12758721; doi:10.1371/journal.pntd.0013840)
Supplement: S3 Table — The table shows the limit of detection at nine serial dilutions (108-100 CFU/ml). (DOCX) [file pntd.0013840.s004.docx]

| *B. pseudomallei* concentration (CFU/ml) | Mean Ct value cycle (SD) | |
| --- | --- | --- |
|  | *BPSS1187*-PCR | *TTS1-orf2*-PCR |
| 1x10^8^ | 15.75 (0.09) | 13.72 (0.04) |
| 1x10^7^ | 19.23 (0.30) | 17.17 (0.16) |
| 1x10^6^ | 22.59 (0.45) | 21.80 (0.57) |
| 1x10^5^ | 26.57 (0.55) | 30.73 (0.38) |
| 1x10^4^ | 29.31 (0.17) | 34.30 (1.02) |
| 1x10^3^ | 34.64 (0.47) | - |
| 1x10^2^ | 35.10 (1.63) | - |
| 1x10^1^ | - | - |
| 1x10^0^ | - | - |

**S3 Table. Limit of detection (LOD) of real-time PCR targeting *BPSS1187* and *TTS1-orf2* genes in *B. pseudomallei* K96243-spiked PBS.** The table shows the limit of detection at nine serial dilutions (10^8^-10^0^ CFU/ml)
