# Supplementary material for: Evaluation of culture- and PCR-based methods for detecting Burkholderia pseudomallei in soil samples in Thailand
Source: PLoS Negl Trop Dis. 2026 Jan 2;20(1):e0013840. doi: 10.1371/journal.pntd.0013840 (PMC12758721; doi:10.1371/journal.pntd.0013840)
Supplement: S4 Table — The table shows the regression equations, correlation coefficients (R2 values), and amplification efficiencies for each gene target. (DOCX) [file pntd.0013840.s005.docx]

**S4 Table. Linear regression analysis for real-time PCR targeting *BPSS1187* and**

***TTS1-orf2* genes.** The table shows the regression equations, correlation coefficients (R² values), and amplification efficiencies for each gene target.

| ***B. pseudomallei* strains** | **Equations** | **Correlation coefficients (R^2^)** | **PCR efficiency (%)** |
| --- | --- | --- | --- |
| ***BPSS1187*-PCR** | | | |
| 30-191-S08 | y = -3.808x + 49.45 | 0.9970 | 83.06 |
| 30-191-S10 | y = -3.409x + 49.78 | 0.9601 | 96.49 |
| 30-191-S16 | y = -3.489x + 45.90 | 0.9861 | 93.47 |
| 30-191-S17 | y = -3.459x + 51.72 | 0.9469 | 94.58 |
| 30-194-S03 | y = -4.007x + 49.78 | 0.9940 | 77.64 |
| 30-194-S04 | y = -4.047x + 50.66 | 0.9977 | 76.64 |
| 30-194-S14 | y = -4.022x + 55.53 | 0.9879 | 77.26 |
| 30-198-S22 | y = -3.579x + 47.19 | 0.9886 | 90.28 |
| 30-198-S23 | y = -4.065x + 50.85 | 0.9938 | 76.19 |
| 30-198-S28 | y = -3.705x + 53.65 | 0.9670 | 86.16 |
| ***TTS1-orf2*-PCR** | | | |
| 30-191-S08 | y = -3.928x + 48.60 | 0.9991 | 79.71 |
| 30-191-S10 | y = -3.009x + 44.75 | 0.6819 | 114.94 |
| 30-191-S16 | y = -2.847x + 42.95 | 0.9561 | 124.51 |
| 30-191-S17 | y = -3.517x + 52.25 | 0.9475 | 92.45 |
| 30-194-S03 | y = -3.541x + 46.64 | 0.9979 | 91.60 |
| 30-194-S04 | y = -4.203x + 50.19 | 0.9978 | 72.95 |
| 30-194-S14 | y = -3.535x + 50.35 | 0.9900 | 91.81 |
| 30-198-S22 | y = -3.839x + 47.49 | 0.9954 | 82.17 |
| 30-198-S23 | y = -3.801x + 47.21 | 0.9937 | 83.26 |
| 30-198-S28 | y = -3.999x + 52.48 | 0.9733 | 77.85 |
