# Supplementary material for: Evaluation of culture- and PCR-based methods for detecting Burkholderia pseudomallei in soil samples in Thailand
Source: PLoS Negl Trop Dis. 2026 Jan 2;20(1):e0013840. doi: 10.1371/journal.pntd.0013840 (PMC12758721; doi:10.1371/journal.pntd.0013840)
Supplement: S5 Table — The table shows the limit of detection at nine serial dilutions (108-100 CFU/ml). (DOCX) [file pntd.0013840.s006.docx]

**S5 Table. Limit of detection (LOD) of real-time PCR targeting *BPSS1187* and *TTS1-orf2* genes in spiked soil with ten environmental *B. pseudomallei* strains.** The table shows the limit of detection at nine serial dilutions (10^8^-10^0^ CFU/ml)

***BPSS1187*-PCR**

| *B. pseudomallei* concentration (CFU/ml) | Environmental *B. pseudomallei* strains | | | | | | | | | |
| --- | --- | --- | --- | --- | --- | --- | --- | --- | --- | --- |
|  | 30-191-S08 | 30-191-S10 | 30-191-S16 | 30-191-S17 | 30-194-S03 | 30-194-S04 | 30-194-S14 | 30-198-S22 | 30-198-S23 | 30-198-S28 |
| 1x10^8^ | 18.71 | 23.53 | 18.7 | 23.29 | 17.87 | 18.45 | 23.05 | 18.26 | 18.37 | 22.8 |
| 1x10^7^ | 22.83 | 24.19 | 21.02 | 27.12 | 21.82 | 22.44 | 27.32 | 22.42 | 22.31 | 28.53 |
| 1x10^6^ | 27.16 | 29.47 | 24.07 | 33.26 | 25.68 | 25.99 | 31.56 | 25.41 | 26.08 | 32.26 |
| 1x10^5^ | 30.31 | 33.58 | 28.75 | 34.03 | 28.97 | 30.17 | 36.48 | 30.21 | 31.36 | 35.84 |
| 1x10^4^ | 34.01 | 35.88 | 32.28 | 37.13 | 34.33 | 34.82 | 38.58 | 32.26 | 34.17 | 37.67 |
| 1x10^3^ | - | - | - | - | - | - | - | - | - | - |
| 1x10^2^ | - | - | - | - | - | - | - | - | - | - |
| 1x10^1^ | - | - | - | - | - | - | - | - | - | - |
| 1x10^0^ | - | - | - | - | - | - | - | - | - | - |

***TTS1-orf2*-PCR**

| *B. pseudomallei* concentration (CFU/ml) | Environmental *B. pseudomallei* strains | | | | | | | | | |
| --- | --- | --- | --- | --- | --- | --- | --- | --- | --- | --- |
|  | 30-191-S08 | 30-191-S10 | 30-191-S16 | 30-191-S17 | 30-194-S03 | 30-194-S04 | 30-194-S14 | 30-198-S22 | 30-198-S23 | 30-198-S28 |
| 1x10^8^ | 17.33 | 20.66 | 18.95 | 23.25 | 18.13 | 16.7 | 21.81 | 16.59 | 16.55 | 19.47 |
| 1x10^7^ | 21.11 | 26.86 | 24.22 | 27.3 | 21.83 | 20.96 | 25.34 | 20.78 | 20.78 | 24.99 |
| 1x10^6^ | 24.73 | 21.27 | 26.5 | 33.36 | 25.71 | 24.45 | 30.14 | 24.28 | 24.28 | 29.32 |
| 1x10^5^ | 28.97 | 31.11 | 28.71 | 34.71 | 29.14 | 29.13 | 32.53 | 28.93 | 28.93 | 33.42 |
| 1x10^4^ | 33.04 | 33.58 | 30.94 | 37.13 | 32.18 | 33.63 | 35.89 | 31.71 | 31.48 | 35.25 |
| 1x10^3^ | - | - | - | - | - | - | - | - | - | - |
| 1x10^2^ | - | - | - | - | - | - | - | - | - | - |
| 1x10^1^ | - | - | - | - | - | - | - | - | - | - |
| 1x10^0^ | - | - | - | - | - | - | - | - | - | - |
