# Supplementary material for: Evaluation of culture- and PCR-based methods for detecting Burkholderia pseudomallei in soil samples in Thailand
Source: PLoS Negl Trop Dis. 2026 Jan 2;20(1):e0013840. doi: 10.1371/journal.pntd.0013840 (PMC12758721; doi:10.1371/journal.pntd.0013840)
Supplement: S6 Table — The table presents seroprevalence data, with the first cut-off value (0.418) highlighted in bold purple and the second cut-off value (1.165) highlighted in bold red. (DOCX) [file pntd.0013840.s007.docx]

**S6 Table. Seroprevalence data of healthy individuals in Amnat Charoen Province.** The table presents seroprevalence data, with the first cut-off value (0.418) highlighted in **bold purple** and the second cut-off value (1.165) highlighted in **bold red.**

|  | | | | | | | | |
| --- | --- | --- | --- | --- | --- | --- | --- | --- |
| **Mueang Amnat Charoen district** | | | | | | | | |
| **Sample Number** | **Sample ID** | **OD1** | **OD2** | **OD Average** | **Assay diluent 1** | **Assay diluent 2** | **Average** | **Analyzed** |
| 1 | A-1-1 | 0.122 | 0.118 | 0.12 | 0.068 | 0.071 | 0.0695 | 0.0505 |
| 2 | A-1-2 | 0.198 | 0.202 | 0.2 | 0.068 | 0.071 | 0.0695 | 0.1305 |
| 3 | A-1-3 | 0.248 | 0.249 | 0.2485 | 0.068 | 0.071 | 0.0695 | 0.179 |
| 4 | A-1-4 | 0.091 | 0.089 | 0.09 | 0.068 | 0.071 | 0.0695 | 0.0205 |
| 5 | A-1-5 | 0.08 | 0.08 | 0.08 | 0.068 | 0.071 | 0.0695 | 0.0105 |
| 6 | A-1-6 | 0.187 | 0.187 | 0.187 | 0.068 | 0.071 | 0.0695 | 0.1175 |
| 7 | A-1-7 | 0.095 | 0.091 | 0.093 | 0.068 | 0.071 | 0.0695 | 0.0235 |
| 8 | A-1-8 | 0.102 | 0.097 | 0.0995 | 0.068 | 0.071 | 0.0695 | 0.03 |
| **9** | **A-1-9** | **2.067** | **1.904** | **1.9855** | **0.068** | **0.071** | **0.0695** | **1.916** |
| 10 | A-1-10 | 0.234 | 0.237 | 0.2355 | 0.068 | 0.071 | 0.0695 | 0.166 |
| 11 | A-1-11 | 0.144 | 0.143 | 0.1435 | 0.068 | 0.071 | 0.0695 | 0.074 |
| 12 | A-1-12 | 0.105 | 0.11 | 0.1075 | 0.068 | 0.071 | 0.0695 | 0.038 |
| **13** | **A-1-16** | **3.205** | **3.2** | **3.2025** | **0.068** | **0.071** | **0.0695** | **3.133** |
| 14 | A-1-18 | 0.336 | 0.333 | 0.3345 | 0.068 | 0.071 | 0.0695 | 0.265 |
| 15 | A-1-19 | 0.133 | 0.135 | 0.134 | 0.068 | 0.071 | 0.0695 | 0.0645 |
| 16 | A-2-1 | 0.104 | 0.101 | 0.1025 | 0.068 | 0.071 | 0.0695 | 0.033 |
| 17 | A-2-2 | 0.1 | 0.1 | 0.1 | 0.068 | 0.071 | 0.0695 | 0.0305 |
| 18 | A-2-3 | 0.118 | 0.118 | 0.118 | 0.068 | 0.071 | 0.0695 | 0.0485 |
| **19** | **A-2-4** | **0.793** | **0.828** | **0.8105** | **0.068** | **0.071** | **0.0695** | **0.741** |
| 20 | A-2-5 | 0.113 | 0.117 | 0.115 | 0.068 | 0.071 | 0.0695 | 0.0455 |
| 21 | A-2-6 | 0.14 | 0.139 | 0.1395 | 0.068 | 0.071 | 0.0695 | 0.07 |
| 22 | A-2-7 | 0.089 | 0.091 | 0.09 | 0.068 | 0.071 | 0.0695 | 0.0205 |
| 23 | A-2-8 | 0.087 | 0.087 | 0.087 | 0.068 | 0.071 | 0.0695 | 0.0175 |
| **24** | **A-2-9** | **0.585** | **0.593** | **0.589** | **0.068** | **0.071** | **0.0695** | **0.5195** |
| 25 | A-2-10 | 0.109 | 0.109 | 0.109 | 0.068 | 0.071 | 0.0695 | 0.0395 |
| 26 | A-2-11 | 0.162 | 0.163 | 0.1625 | 0.068 | 0.071 | 0.0695 | 0.093 |
| 27 | A-2-12 | 0.16 | 0.161 | 0.1605 | 0.068 | 0.071 | 0.0695 | 0.091 |
| 28 | A-2-13 | 0.093 | 0.098 | 0.0955 | 0.068 | 0.071 | 0.0695 | 0.026 |
| 29 | A-2-14 | 0.095 | 0.093 | 0.094 | 0.068 | 0.071 | 0.0695 | 0.0245 |
| 30 | A-2-15 | 0.085 | 0.084 | 0.0845 | 0.068 | 0.071 | 0.0695 | 0.015 |
| 31 | A-2-16 | 0.191 | 0.203 | 0.197 | 0.068 | 0.071 | 0.0695 | 0.1275 |
| 32 | A-2-17 | 0.079 | 0.08 | 0.0795 | 0.068 | 0.071 | 0.0695 | 0.01 |
| 33 | A-2-18 | 0.136 | 0.138 | 0.137 | 0.068 | 0.071 | 0.0695 | 0.0675 |
| 34 | A-2-19 | 0.114 | 0.111 | 0.1125 | 0.068 | 0.071 | 0.0695 | 0.043 |
| 35 | A-2-20 | 0.097 | 0.095 | 0.096 | 0.068 | 0.071 | 0.0695 | 0.0265 |
| 36 | A-2-21 | 0.089 | 0.094 | 0.0915 | 0.068 | 0.071 | 0.0695 | 0.022 |
| 37 | A-2-22 | 0.232 | 0.246 | 0.239 | 0.068 | 0.071 | 0.0695 | 0.1695 |
| 38 | A-2-23 | 0.25 | 0.244 | 0.247 | 0.068 | 0.071 | 0.0695 | 0.1775 |
| 39 | A-2-24 | 0.108 | 0.108 | 0.108 | 0.068 | 0.071 | 0.0695 | 0.0385 |
| 40 | A-2-25 | 0.38 | 0.398 | 0.389 | 0.068 | 0.071 | 0.0695 | 0.3195 |
| 41 | A-2-26 | 0.089 | 0.088 | 0.0885 | 0.068 | 0.071 | 0.0695 | 0.019 |
| 42 | A-2-27 | 0.113 | 0.103 | 0.108 | 0.068 | 0.071 | 0.0695 | 0.0385 |
| 43 | A-2-28 | 0.158 | 0.165 | 0.1615 | 0.068 | 0.071 | 0.0695 | 0.092 |
| 44 | A-2-29 | 0.094 | 0.092 | 0.093 | 0.068 | 0.071 | 0.0695 | 0.0235 |
| 45 | A-2-30 | 0.253 | 0.251 | 0.252 | 0.068 | 0.071 | 0.0695 | 0.1825 |
| 46 | A-2-31 | 0.093 | 0.09 | 0.0915 | 0.061 | 0.062 | 0.0615 | 0.03 |
| **47** | **A-2-32** | **0.737** | **0.562** | **0.6495** | **0.061** | **0.062** | **0.0615** | **0.588** |
| 48 | A-2-33 | 0.312 | 0.275 | 0.2935 | 0.061 | 0.062 | 0.0615 | 0.232 |
| 49 | A-2-34 | 0.17 | 0.161 | 0.1655 | 0.061 | 0.062 | 0.0615 | 0.104 |
| 50 | A-2-35 | 0.062 | 0.065 | 0.0635 | 0.061 | 0.062 | 0.0615 | 0.002 |
| 51 | A-2-36 | 0.101 | 0.096 | 0.0985 | 0.061 | 0.062 | 0.0615 | 0.037 |
| 52 | A-2-37 | 0.074 | 0.071 | 0.0725 | 0.061 | 0.062 | 0.0615 | 0.011 |
| 53 | A-2-38 | 0.092 | 0.09 | 0.091 | 0.061 | 0.062 | 0.0615 | 0.0295 |
| 54 | A-2-39 | 0.094 | 0.093 | 0.0935 | 0.061 | 0.062 | 0.0615 | 0.032 |
| 55 | A-2-40 | 0.074 | 0.071 | 0.0725 | 0.061 | 0.062 | 0.0615 | 0.011 |
| 56 | A-2-41 | 0.179 | 0.159 | 0.169 | 0.061 | 0.062 | 0.0615 | 0.1075 |
| 57 | A-2-42 | 0.122 | 0.122 | 0.122 | 0.061 | 0.062 | 0.0615 | 0.0605 |
| 58 | A-2-43 | 0.35 | 0.413 | 0.3815 | 0.061 | 0.062 | 0.0615 | 0.32 |
| 59 | A-2-44 | 0.078 | 0.078 | 0.078 | 0.061 | 0.062 | 0.0615 | 0.0165 |
| **60** | **A-2-45** | **0.766** | **0.733** | **0.7495** | **0.061** | **0.062** | **0.0615** | **0.688** |
| 61 | A-2-46 | 0.077 | 0.075 | 0.076 | 0.061 | 0.062 | 0.0615 | 0.0145 |
| 62 | A-2-48 | 0.067 | 0.066 | 0.0665 | 0.061 | 0.062 | 0.0615 | 0.005 |
| 63 | A-2-49 | 0.088 | 0.09 | 0.089 | 0.061 | 0.062 | 0.0615 | 0.0275 |
| 64 | A-2-50 | 0.174 | 0.181 | 0.1775 | 0.061 | 0.062 | 0.0615 | 0.116 |
| 65 | A-2-51 | 0.166 | 0.167 | 0.1665 | 0.061 | 0.062 | 0.0615 | 0.105 |
| 66 | A-2-52 | 0.102 | 0.091 | 0.0965 | 0.061 | 0.062 | 0.0615 | 0.035 |
| **67** | **A-2-53** | **0.642** | **0.561** | **0.6015** | **0.061** | **0.062** | **0.0615** | **0.54** |
| 68 | A-2-54 | 0.135 | 0.134 | 0.1345 | 0.061 | 0.062 | 0.0615 | 0.073 |
| 69 | A-2-55 | 0.139 | 0.133 | 0.136 | 0.061 | 0.062 | 0.0615 | 0.0745 |
| 70 | A-2-56 | 0.315 | 0.318 | 0.3165 | 0.061 | 0.062 | 0.0615 | 0.255 |
| 71 | A-2-57 | 0.195 | 0.192 | 0.1935 | 0.061 | 0.062 | 0.0615 | 0.132 |
| 72 | A-2-58 | 0.078 | 0.079 | 0.0785 | 0.061 | 0.062 | 0.0615 | 0.017 |
| 73 | A-2-59 | 0.145 | 0.146 | 0.1455 | 0.061 | 0.062 | 0.0615 | 0.084 |
| 74 | A-2-60 | 0.083 | 0.083 | 0.083 | 0.061 | 0.062 | 0.0615 | 0.0215 |
| 75 | A-2-61 | 0.096 | 0.098 | 0.097 | 0.061 | 0.062 | 0.0615 | 0.0355 |
| 76 | A-2-62 | 0.125 | 0.068 | 0.0965 | 0.061 | 0.062 | 0.0615 | 0.035 |
| 77 | A-2-63 | 0.076 | 0.103 | 0.0895 | 0.061 | 0.062 | 0.0615 | 0.028 |
| 78 | A-2-65 | 0.142 | 0.135 | 0.1385 | 0.061 | 0.062 | 0.0615 | 0.077 |
| 79 | A-2-66 | 0.075 | 0.073 | 0.074 | 0.061 | 0.062 | 0.0615 | 0.0125 |
| 80 | A-2-67 | 0.114 | 0.115 | 0.1145 | 0.061 | 0.062 | 0.0615 | 0.053 |
| 81 | A-2-68 | 0.316 | 0.305 | 0.3105 | 0.061 | 0.062 | 0.0615 | 0.249 |
| **82** | **A-2-69** | **0.603** | **0.566** | **0.5845** | **0.061** | **0.062** | **0.0615** | **0.523** |
| 83 | A-2-70 | 0.334 | 0.313 | 0.3235 | 0.061 | 0.062 | 0.0615 | 0.262 |
| 84 | A-2-71 | 0.201 | 0.219 | 0.21 | 0.061 | 0.062 | 0.0615 | 0.1485 |
| 85 | A-2-72 | 0.068 | 0.07 | 0.069 | 0.061 | 0.062 | 0.0615 | 0.0075 |
| 86 | A-2-73 | 0.065 | 0.068 | 0.0665 | 0.061 | 0.062 | 0.0615 | 0.005 |
| 87 | A-2-74 | 0.073 | 0.079 | 0.076 | 0.061 | 0.062 | 0.0615 | 0.0145 |
| 88 | A-2-75 | 0.087 | 0.094 | 0.0905 | 0.061 | 0.062 | 0.0615 | 0.029 |
| 89 | A-2-76 | 0.126 | 0.127 | 0.1265 | 0.061 | 0.062 | 0.0615 | 0.065 |
| 90 | A-2-77 | 0.174 | 0.201 | 0.1875 | 0.061 | 0.062 | 0.0615 | 0.126 |
| 91 | A-2-78 | 0.153 | 0.146 | 0.1495 | 0.061 | 0.062 | 0.0615 | 0.088 |
| 92 | A-2-79 | 0.08 | 0.081 | 0.0805 | 0.061 | 0.062 | 0.0615 | 0.019 |
| 93 | A-2-80 | 0.077 | 0.08 | 0.0785 | 0.061 | 0.062 | 0.0615 | 0.017 |
| 94 | A-2-81 | 0.113 | 0.111 | 0.112 | 0.061 | 0.062 | 0.0615 | 0.0505 |
| 95 | A-2-82 | 0.113 | 0.107 | 0.11 | 0.061 | 0.062 | 0.0615 | 0.0485 |
| 96 | A-2-83 | 0.152 | 0.154 | 0.153 | 0.061 | 0.062 | 0.0615 | 0.0915 |
| 97 | A-2-84 | 0.103 | 0.102 | 0.1025 | 0.061 | 0.062 | 0.0615 | 0.041 |
| 98 | A-2-85 | 0.146 | 0.149 | 0.1475 | 0.061 | 0.062 | 0.0615 | 0.086 |
| 99 | A-2-86 | 0.124 | 0.136 | 0.13 | 0.061 | 0.062 | 0.0615 | 0.0685 |
| 100 | A-2-87 | 0.1 | 0.096 | 0.098 | 0.061 | 0.062 | 0.0615 | 0.0365 |
| 101 | A-2-88 | 0.079 | 0.079 | 0.079 | 0.061 | 0.062 | 0.0615 | 0.0175 |
| 102 | A-2-89 | 0.098 | 0.094 | 0.096 | 0.061 | 0.062 | 0.0615 | 0.0345 |
| 103 | A-2-90 | 0.087 | 0.084 | 0.0855 | 0.061 | 0.062 | 0.0615 | 0.024 |
| 104 | A-2-91 | 0.104 | 0.109 | 0.1065 | 0.061 | 0.062 | 0.0615 | 0.045 |
| 105 | A-2-92 | 0.081 | 0.082 | 0.0815 | 0.061 | 0.062 | 0.0615 | 0.02 |
| 106 | A-3-1 | 0.07 | 0.072 | 0.071 | 0.061 | 0.062 | 0.0615 | 0.0095 |
| 107 | A-3-2 | 0.106 | 0.11 | 0.108 | 0.061 | 0.062 | 0.0615 | 0.0465 |
| 108 | A-3-3 | 0.226 | 0.207 | 0.2165 | 0.061 | 0.062 | 0.0615 | 0.155 |
| 109 | A-3-4 | 0.125 | 0.125 | 0.125 | 0.061 | 0.062 | 0.0615 | 0.0635 |
| 110 | A-3-5 | 0.109 | 0.112 | 0.1105 | 0.061 | 0.062 | 0.0615 | 0.049 |
| 111 | A-3-6 | 0.121 | 0.121 | 0.121 | 0.061 | 0.062 | 0.0615 | 0.0595 |
| 112 | A-3-7 | 0.079 | 0.082 | 0.0805 | 0.061 | 0.062 | 0.0615 | 0.019 |
| 113 | A-3-8 | 0.087 | 0.089 | 0.088 | 0.061 | 0.062 | 0.0615 | 0.0265 |
| 114 | A-3-9 | 0.074 | 0.075 | 0.0745 | 0.061 | 0.062 | 0.0615 | 0.013 |
| 115 | A-3-10 | 0.077 | 0.077 | 0.077 | 0.061 | 0.062 | 0.0615 | 0.0155 |
| 116 | A-3-11 | 0.258 | 0.252 | 0.255 | 0.061 | 0.062 | 0.0615 | 0.1935 |
| 117 | A-3-12 | 0.08 | 0.077 | 0.0785 | 0.061 | 0.062 | 0.0615 | 0.017 |
| 118 | A-3-13 | 0.075 | 0.074 | 0.0745 | 0.061 | 0.062 | 0.0615 | 0.013 |
| 119 | A-3-14 | 0.154 | 0.155 | 0.1545 | 0.061 | 0.062 | 0.0615 | 0.093 |
| 120 | A-3-15 | 0.095 | 0.099 | 0.097 | 0.061 | 0.062 | 0.0615 | 0.0355 |
| 121 | A-3-16 | 0.144 | 0.147 | 0.1455 | 0.061 | 0.062 | 0.0615 | 0.084 |
| 122 | A-3-17 | 0.152 | 0.158 | 0.155 | 0.061 | 0.062 | 0.0615 | 0.0935 |
| 123 | A-3-18 | 0.125 | 0.12 | 0.1225 | 0.061 | 0.062 | 0.0615 | 0.061 |
| 124 | A-3-19 | 0.097 | 0.094 | 0.0955 | 0.061 | 0.062 | 0.0615 | 0.034 |
| 125 | A-3-20 | 0.144 | 0.138 | 0.141 | 0.061 | 0.062 | 0.0615 | 0.0795 |
| 126 | A-3-21 | 0.093 | 0.091 | 0.092 | 0.061 | 0.062 | 0.0615 | 0.0305 |
| 127 | A-3-22 | 0.079 | 0.079 | 0.079 | 0.061 | 0.062 | 0.0615 | 0.0175 |
| 128 | A-3-23 | 0.082 | 0.082 | 0.082 | 0.061 | 0.062 | 0.0615 | 0.0205 |
| 129 | A-3-24 | 0.089 | 0.092 | 0.0905 | 0.061 | 0.062 | 0.0615 | 0.029 |
| 130 | A-3-25 | 0.072 | 0.077 | 0.0745 | 0.061 | 0.062 | 0.0615 | 0.013 |
| 131 | A-3-26 | 0.097 | 0.101 | 0.099 | 0.061 | 0.062 | 0.0615 | 0.0375 |
| **132** | **A-3-27** | **3.357** | **3.305** | **3.331** | **0.061** | **0.062** | **0.0615** | **3.2695** |
| 133 | A-3-28 | 0.224 | 0.247 | 0.2355 | 0.061 | 0.062 | 0.0615 | 0.174 |
| 134 | A-3-29 | 0.245 | 0.222 | 0.2335 | 0.061 | 0.062 | 0.0615 | 0.172 |
| 135 | A-3-30 | 0.251 | 0.244 | 0.2475 | 0.061 | 0.062 | 0.0615 | 0.186 |
| 136 | A-3-31 | 0.122 | 0.121 | 0.1215 | 0.061 | 0.062 | 0.0615 | 0.06 |
| **Hua Taphan** | | | | | | | | |
| **Sample Number** | **Sample ID** | **OD1** | **OD2** | **OD Average** | **Assay diluent 1** | **Assay diluent 2** | **Average** | **Analyzed** |
| 1 | B-1-1 | 0.109 | 0.108 | 0.1085 | 0.061 | 0.062 | 0.0615 | 0.047 |
| 2 | B-1-2 | 0.086 | 0.084 | 0.085 | 0.061 | 0.062 | 0.0615 | 0.0235 |
| 3 | B-1-3 | 0.095 | 0.091 | 0.093 | 0.061 | 0.062 | 0.0615 | 0.0315 |
| 4 | B-1-4 | 0.098 | 0.105 | 0.1015 | 0.061 | 0.062 | 0.0615 | 0.04 |
| 5 | B-1-5 | 0.375 | 0.432 | 0.4035 | 0.061 | 0.062 | 0.0615 | 0.342 |
| 6 | B-1-6 | 0.085 | 0.08 | 0.0825 | 0.061 | 0.062 | 0.0615 | 0.021 |
| 7 | B-1-7 | 0.065 | 0.064 | 0.0645 | 0.061 | 0.062 | 0.0615 | 0.003 |
| 8 | B-2-1 | 0.077 | 0.077 | 0.077 | 0.061 | 0.062 | 0.0615 | 0.0155 |
| 9 | B-2-2 | 0.092 | 0.098 | 0.095 | 0.061 | 0.062 | 0.0615 | 0.0335 |
| 10 | B-2-3 | 0.547 | 0.552 | 0.5495 | 0.061 | 0.062 | 0.0615 | 0.488 |
| 11 | B-2-4 | 0.107 | 0.103 | 0.105 | 0.061 | 0.062 | 0.0615 | 0.0435 |
| 12 | B-2-5 | 0.092 | 0.122 | 0.107 | 0.061 | 0.062 | 0.0615 | 0.0455 |
| 13 | B-2-6 | 0.103 | 0.097 | 0.1 | 0.061 | 0.062 | 0.0615 | 0.0385 |
| 14 | B-2-7 | 0.08 | 0.077 | 0.0785 | 0.061 | 0.062 | 0.0615 | 0.017 |
| 15 | B-2-8 | 0.15 | 0.139 | 0.1445 | 0.061 | 0.062 | 0.0615 | 0.083 |
| 16 | B-2-9 | 0.109 | 0.204 | 0.1565 | 0.061 | 0.062 | 0.0615 | 0.095 |
| 17 | B-2-10 | 0.081 | 0.08 | 0.0805 | 0.061 | 0.062 | 0.0615 | 0.019 |
| 18 | B-2-11 | 0.147 | 0.14 | 0.1435 | 0.061 | 0.062 | 0.0615 | 0.082 |
| 19 | B-2-12 | 0.062 | 0.061 | 0.0615 | 0.061 | 0.062 | 0.0615 | 0 |
| 20 | B-2-13 | 0.084 | 0.083 | 0.0835 | 0.061 | 0.062 | 0.0615 | 0.022 |
| 21 | B-2-14 | 0.461 | 0.453 | 0.457 | 0.061 | 0.062 | 0.0615 | 0.3955 |
| 22 | B-2-15 | 0.285 | 0.305 | 0.295 | 0.061 | 0.062 | 0.0615 | 0.2335 |
| 23 | B-2-16 | 0.121 | 0.116 | 0.1185 | 0.061 | 0.062 | 0.0615 | 0.057 |
| 24 | B-2-17 | 0.1 | 0.099 | 0.0995 | 0.061 | 0.062 | 0.0615 | 0.038 |
| 25 | B-2-18 | 0.517 | 0.536 | 0.5265 | 0.061 | 0.062 | 0.0615 | 0.465 |
| 26 | B-2-19 | 0.222 | 0.202 | 0.212 | 0.061 | 0.062 | 0.0615 | 0.1505 |
| 27 | B-2-20 | 0.522 | 0.501 | 0.5115 | 0.061 | 0.062 | 0.0615 | 0.45 |
| 28 | B-2-21 | 0.08 | 0.082 | 0.081 | 0.061 | 0.062 | 0.0615 | 0.0195 |
| 29 | B-2-22 | 0.274 | 0.272 | 0.273 | 0.061 | 0.062 | 0.0615 | 0.2115 |
| 30 | B-2-23 | 0.074 | 0.076 | 0.075 | 0.061 | 0.062 | 0.0615 | 0.0135 |
| 31 | B-2-24 | 0.062 | 0.069 | 0.0655 | 0.061 | 0.062 | 0.0615 | 0.004 |
| 32 | B-2-25 | 0.086 | 0.088 | 0.087 | 0.061 | 0.062 | 0.0615 | 0.0255 |
| 33 | B-2-26 | 0.082 | 0.083 | 0.0825 | 0.061 | 0.062 | 0.0615 | 0.021 |
| 34 | B-2-27 | 0.096 | 0.097 | 0.0965 | 0.061 | 0.062 | 0.0615 | 0.035 |
| 35 | B-2-28 | 0.122 | 0.123 | 0.1225 | 0.061 | 0.062 | 0.0615 | 0.061 |
| 36 | B-2-29 | 0.111 | 0.106 | 0.1085 | 0.061 | 0.062 | 0.0615 | 0.047 |
| 37 | B-2-30 | 0.093 | 0.094 | 0.0935 | 0.061 | 0.062 | 0.0615 | 0.032 |
| 38 | B-2-31 | 0.08 | 0.085 | 0.0825 | 0.061 | 0.062 | 0.0615 | 0.021 |
| 39 | B-2-32 | 0.162 | 0.168 | 0.165 | 0.061 | 0.062 | 0.0615 | 0.1035 |
| 40 | B-2-33 | 0.078 | 0.076 | 0.077 | 0.061 | 0.062 | 0.0615 | 0.0155 |
| 41 | B-3-1 | 0.087 | 0.083 | 0.085 | 0.061 | 0.062 | 0.0615 | 0.0235 |
| 42 | B-3-2 | 0.106 | 0.111 | 0.1085 | 0.061 | 0.062 | 0.0615 | 0.047 |
| 43 | B-3-3 | 0.086 | 0.082 | 0.084 | 0.061 | 0.062 | 0.0615 | 0.0225 |
| 44 | B-3-4 | 0.083 | 0.085 | 0.084 | 0.061 | 0.062 | 0.0615 | 0.0225 |
| **Lue Amnat district** | | | | | | | | |
| **Sample Number** | **Sample ID** | **OD1** | **OD2** | **OD Average** | **Assay diluent 1** | **Assay diluent 2** | **Average** | **Analyzed** |
| 1 | C-1-1 | 0.087 | 0.081 | 0.084 | 0.062 | 0.059 | 0.0605 | 0.0235 |
| 2 | C-1-2 | 0.415 | 0.389 | 0.402 | 0.062 | 0.059 | 0.0605 | 0.3415 |
| 3 | C-1-3 | 0.082 | 0.118 | 0.1 | 0.062 | 0.059 | 0.0605 | 0.0395 |
| **4** | **C-1-4** | **0.775** | **0.725** | **0.75** | **0.062** | **0.059** | **0.0605** | **0.6895** |
| 5 | C-1-5 | 0.151 | 0.142 | 0.1465 | 0.062 | 0.059 | 0.0605 | 0.086 |
| 6 | C-2-1 | 0.094 | 0.091 | 0.0925 | 0.062 | 0.059 | 0.0605 | 0.032 |
| 7 | C-2-2 | 0.215 | 0.208 | 0.2115 | 0.062 | 0.059 | 0.0605 | 0.151 |
| 8 | C-2-3 | 0.077 | 0.077 | 0.077 | 0.062 | 0.059 | 0.0605 | 0.0165 |
| 9 | C-2-4 | 0.085 | 0.088 | 0.0865 | 0.062 | 0.059 | 0.0605 | 0.026 |
| 10 | C-2-5 | 0.132 | 0.133 | 0.1325 | 0.062 | 0.059 | 0.0605 | 0.072 |
| 11 | C-2-6 | 0.188 | 0.196 | 0.192 | 0.062 | 0.059 | 0.0605 | 0.1315 |
| 12 | C-2-7 | 0.105 | 0.103 | 0.104 | 0.062 | 0.059 | 0.0605 | 0.0435 |
| 13 | C-2-8 | 0.104 | 0.104 | 0.104 | 0.062 | 0.059 | 0.0605 | 0.0435 |
| 14 | C-2-9 | 0.314 | 0.31 | 0.312 | 0.062 | 0.059 | 0.0605 | 0.2515 |
| 15 | C-2-10 | 0.08 | 0.08 | 0.08 | 0.062 | 0.059 | 0.0605 | 0.0195 |
| 16 | C-2-11 | 0.08 | 0.082 | 0.081 | 0.062 | 0.059 | 0.0605 | 0.0205 |
| 17 | C-2-12 | 0.124 | 0.112 | 0.118 | 0.062 | 0.059 | 0.0605 | 0.0575 |
| 18 | C-2-13 | 0.164 | 0.165 | 0.1645 | 0.062 | 0.059 | 0.0605 | 0.104 |
| 19 | C-2-14 | 0.132 | 0.144 | 0.138 | 0.062 | 0.059 | 0.0605 | 0.0775 |
| 20 | C-2-15 | 0.08 | 0.14 | 0.11 | 0.062 | 0.059 | 0.0605 | 0.0495 |
| 21 | C-2-16 | 0.084 | 0.075 | 0.0795 | 0.062 | 0.059 | 0.0605 | 0.019 |
| 22 | C-2-17 | 0.112 | 0.126 | 0.119 | 0.062 | 0.059 | 0.0605 | 0.0585 |
| 23 | C-2-18 | 0.18 | 0.174 | 0.177 | 0.062 | 0.059 | 0.0605 | 0.1165 |
| **24** | **C-2-19** | **1.023** | **0.98** | **1.0015** | **0.062** | **0.059** | **0.0605** | **0.941** |
| 25 | C-2-20 | 0.466 | 0.448 | 0.457 | 0.062 | 0.059 | 0.0605 | 0.3965 |
| 26 | C-2-21 | 0.097 | 0.087 | 0.092 | 0.062 | 0.059 | 0.0605 | 0.0315 |
| 27 | C-2-22 | 0.081 | 0.079 | 0.08 | 0.062 | 0.059 | 0.0605 | 0.0195 |
| 28 | C-2-23 | 0.108 | 0.112 | 0.11 | 0.062 | 0.059 | 0.0605 | 0.0495 |
| 29 | C-2-24 | 0.254 | 0.252 | 0.253 | 0.062 | 0.059 | 0.0605 | 0.1925 |
| 30 | C-3-1 | 0.077 | 0.078 | 0.0775 | 0.062 | 0.059 | 0.0605 | 0.017 |
| 31 | C-3-2 | 0.164 | 0.167 | 0.1655 | 0.062 | 0.059 | 0.0605 | 0.105 |
| 32 | C-3-3 | 0.184 | 0.264 | 0.224 | 0.062 | 0.059 | 0.0605 | 0.1635 |
| 33 | C-3-4 | 0.086 | 0.091 | 0.0885 | 0.062 | 0.059 | 0.0605 | 0.028 |
| 34 | C-3-5 | 0.16 | 0.165 | 0.1625 | 0.062 | 0.059 | 0.0605 | 0.102 |
| 35 | C-3-6 | 0.089 | 0.09 | 0.0895 | 0.062 | 0.059 | 0.0605 | 0.029 |
| 36 | C-3-7 | 0.077 | 0.079 | 0.078 | 0.062 | 0.059 | 0.0605 | 0.0175 |
| 37 | C-3-8 | 0.087 | 0.09 | 0.0885 | 0.062 | 0.059 | 0.0605 | 0.028 |
| **38** | **C-3-9** | **0.585** | **0.572** | **0.5785** | **0.062** | **0.059** | **0.0605** | **0.518** |
| **Phana district** | | | | | | | | |
| **Sample Number** | **Sample ID** | **OD1** | **OD2** | **OD Average** | **Assay diluent 1** | **Assay diluent 2** | **Average** | **Analyzed** |
| **1** | **D-1-1** | **0.834** | **0.73** | **0.782** | **0.062** | **0.059** | **0.0605** | **0.7215** |
| 2 | D-1-2 | 0.09 | 0.094 | 0.092 | 0.062 | 0.059 | 0.0605 | 0.0315 |
| 3 | D-1-3 | 0.098 | 0.112 | 0.105 | 0.062 | 0.059 | 0.0605 | 0.0445 |
| 4 | D-1-4 | 0.16 | 0.157 | 0.1585 | 0.062 | 0.059 | 0.0605 | 0.098 |
| 5 | D-2-1 | 0.14 | 0.132 | 0.136 | 0.066 | 0.064 | 0.065 | 0.071 |
| 6 | D-2-2 | 0.121 | 0.118 | 0.1195 | 0.066 | 0.064 | 0.065 | 0.0545 |
| 7 | D-2-3 | 0.063 | 0.063 | 0.063 | 0.066 | 0.064 | 0.065 | -0.002 |
| 8 | D-2-4 | 0.092 | 0.088 | 0.09 | 0.066 | 0.064 | 0.065 | 0.025 |
| 9 | D-2-5 | 0.233 | 0.235 | 0.234 | 0.066 | 0.064 | 0.065 | 0.169 |
| **10** | **D-2-6** | **1.007** | **0.913** | **0.96** | **0.066** | **0.064** | **0.065** | **0.895** |
| 11 | D-2-7 | 0.268 | 0.271 | 0.2695 | 0.066 | 0.064 | 0.065 | 0.2045 |
| 12 | D-2-8 | 0.221 | 0.218 | 0.2195 | 0.066 | 0.064 | 0.065 | 0.1545 |
| 13 | D-2-9 | 0.078 | 0.075 | 0.0765 | 0.066 | 0.064 | 0.065 | 0.0115 |
| 14 | D-2-10 | 0.111 | 0.109 | 0.11 | 0.066 | 0.064 | 0.065 | 0.045 |
| 15 | D-2-11 | 0.075 | 0.076 | 0.0755 | 0.066 | 0.064 | 0.065 | 0.0105 |
| 16 | D-2-12 | 0.168 | 0.172 | 0.17 | 0.066 | 0.064 | 0.065 | 0.105 |
| 17 | D-2-13 | 0.096 | 0.091 | 0.0935 | 0.066 | 0.064 | 0.065 | 0.0285 |
| 18 | D-2-14 | 0.091 | 0.087 | 0.089 | 0.066 | 0.064 | 0.065 | 0.024 |
| 19 | D-2-15 | 0.068 | 0.067 | 0.0675 | 0.066 | 0.064 | 0.065 | 0.0025 |
| 20 | D-2-16 | 0.187 | 0.176 | 0.1815 | 0.066 | 0.064 | 0.065 | 0.1165 |
| 21 | D-2-17 | 0.1 | 0.097 | 0.0985 | 0.066 | 0.064 | 0.065 | 0.0335 |
| 22 | D-3-1 | 0.452 | 0.446 | 0.449 | 0.066 | 0.064 | 0.065 | 0.384 |
| 23 | D-3-2 | 0.155 | 0.147 | 0.151 | 0.066 | 0.064 | 0.065 | 0.086 |
| **24** | **D-3-3** | **0.967** | **1.036** | **1.0015** | **0.066** | **0.064** | **0.065** | **0.9365** |
| 25 | D-3-4 | 0.091 | 0.092 | 0.0915 | 0.066 | 0.064 | 0.065 | 0.0265 |
| 26 | D-3-5 | 0.084 | 0.085 | 0.0845 | 0.066 | 0.064 | 0.065 | 0.0195 |
| 27 | D-3-6 | 0.14 | 0.135 | 0.1375 | 0.066 | 0.064 | 0.065 | 0.0725 |
| 28 | D-3-7 | 0.092 | 0.087 | 0.0895 | 0.066 | 0.064 | 0.065 | 0.0245 |
| **29** | **D-3-8** | **0.56** | **0.677** | **0.6185** | **0.066** | **0.064** | **0.065** | **0.5535** |
| 30 | D-3-9 | 0.11 | 0.113 | 0.1115 | 0.066 | 0.064 | 0.065 | 0.0465 |
| 31 | D-3-10 | 0.066 | 0.07 | 0.068 | 0.066 | 0.064 | 0.065 | 0.003 |
| **Pathum Ratchawongsa district** | | | | | | | | |
| **Sample Number** | **Sample ID** | **OD1** | **OD2** | **OD Average** | **Assay diluent 1** | **Assay diluent 2** | **Average** | **Analyzed** |
| 1 | E-1-1 | 0.072 | 0.073 | 0.0725 | 0.066 | 0.064 | 0.065 | 0.0075 |
| 2 | E-1-2 | 0.224 | 0.219 | 0.2215 | 0.066 | 0.064 | 0.065 | 0.1565 |
| 3 | E-1-3 | 0.101 | 0.106 | 0.1035 | 0.066 | 0.064 | 0.065 | 0.0385 |
| 4 | E-1-4 | 0.17 | 0.161 | 0.1655 | 0.066 | 0.064 | 0.065 | 0.1005 |
| 5 | E-1-5 | 0.215 | 0.211 | 0.213 | 0.066 | 0.064 | 0.065 | 0.148 |
| 6 | E-1-6 | 0.083 | 0.085 | 0.084 | 0.066 | 0.064 | 0.065 | 0.019 |
| 7 | E-1-7 | 0.42 | 0.424 | 0.422 | 0.066 | 0.064 | 0.065 | 0.357 |
| 8 | E-2-1 | 0.083 | 0.091 | 0.087 | 0.066 | 0.064 | 0.065 | 0.022 |
| 9 | E-2-2 | 0.105 | 0.104 | 0.1045 | 0.066 | 0.064 | 0.065 | 0.0395 |
| 10 | E-2-3 | 0.212 | 0.22 | 0.216 | 0.066 | 0.064 | 0.065 | 0.151 |
| 11 | E-2-4 | 0.54 | 0.561 | 0.5505 | 0.066 | 0.064 | 0.065 | 0.4855 |
| 12 | E-2-5 | 0.069 | 0.072 | 0.0705 | 0.066 | 0.064 | 0.065 | 0.0055 |
| 13 | E-2-6 | 0.215 | 0.198 | 0.2065 | 0.066 | 0.064 | 0.065 | 0.1415 |
| 14 | E-2-7 | 0.224 | 0.196 | 0.21 | 0.066 | 0.064 | 0.065 | 0.145 |
| 15 | E-2-8 | 0.084 | 0.082 | 0.083 | 0.066 | 0.064 | 0.065 | 0.018 |
| 16 | E-2-9 | 0.076 | 0.079 | 0.0775 | 0.066 | 0.064 | 0.065 | 0.0125 |
| 17 | E-2-10 | 0.188 | 0.167 | 0.1775 | 0.066 | 0.064 | 0.065 | 0.1125 |
| 18 | E-2-11 | 0.099 | 0.098 | 0.0985 | 0.066 | 0.064 | 0.065 | 0.0335 |
| 19 | E-2-12 | 0.225 | 0.207 | 0.216 | 0.084 | 0.089 | 0.0865 | 0.1295 |
| 20 | E-2-13 | 0.123 | 0.118 | 0.1205 | 0.084 | 0.089 | 0.0865 | 0.034 |
| 21 | E-2-14 | 0.144 | 0.141 | 0.1425 | 0.084 | 0.089 | 0.0865 | 0.056 |
| **22** | **E-2-15** | **1.279** | **1.178** | **1.2285** | **0.084** | **0.089** | **0.0865** | **1.142** |
| 23 | E-2-16 | 0.282 | 0.305 | 0.2935 | 0.084 | 0.089 | 0.0865 | 0.207 |
| 24 | E-2-17 | 0.532 | 0.524 | 0.528 | 0.084 | 0.089 | 0.0865 | 0.4415 |
| 25 | E-2-18 | 0.145 | 0.133 | 0.139 | 0.084 | 0.089 | 0.0865 | 0.0525 |
| 26 | E-2-19 | 0.499 | 0.415 | 0.457 | 0.084 | 0.089 | 0.0865 | 0.3705 |
| 27 | E-2-20 | 0.113 | 0.103 | 0.108 | 0.084 | 0.089 | 0.0865 | 0.0215 |
| 28 | E-2-21 | 0.277 | 0.253 | 0.265 | 0.084 | 0.089 | 0.0865 | 0.1785 |
| 29 | E-2-22 | 0.184 | 0.18 | 0.182 | 0.084 | 0.089 | 0.0865 | 0.0955 |
| 30 | E-2-23 | 0.203 | 0.202 | 0.2025 | 0.084 | 0.089 | 0.0865 | 0.116 |
| 31 | E-2-24 | 0.104 | 0.11 | 0.107 | 0.084 | 0.089 | 0.0865 | 0.0205 |
| 32 | E-2-25 | 0.13 | 0.124 | 0.127 | 0.084 | 0.089 | 0.0865 | 0.0405 |
| 33 | E-2-26 | 0.21 | 0.188 | 0.199 | 0.084 | 0.089 | 0.0865 | 0.1125 |
| 34 | E-2-27 | 0.125 | 0.12 | 0.1225 | 0.084 | 0.089 | 0.0865 | 0.036 |
| 35 | E-2-28 | 0.199 | 0.191 | 0.195 | 0.084 | 0.089 | 0.0865 | 0.1085 |
| 36 | E-2-29 | 0.154 | 0.14 | 0.147 | 0.084 | 0.089 | 0.0865 | 0.0605 |
| **37** | **E-2-30** | **1.244** | **1.324** | **1.284** | **0.084** | **0.089** | **0.0865** | **1.1975** |
| 38 | E-2-31 | 0.118 | 0.3 | 0.209 | 0.084 | 0.089 | 0.0865 | 0.1225 |
| 39 | E-3-1 | 0.359 | 0.466 | 0.4125 | 0.084 | 0.089 | 0.0865 | 0.326 |
| 40 | E-3-2 | 0.166 | 0.163 | 0.1645 | 0.084 | 0.089 | 0.0865 | 0.078 |
| 41 | E-3-3 | 0.384 | 0.385 | 0.3845 | 0.084 | 0.089 | 0.0865 | 0.298 |
| 42 | E-3-4 | 0.524 | 0.572 | 0.548 | 0.084 | 0.089 | 0.0865 | 0.4615 |
| 43 | E-3-5 | 0.138 | 0.136 | 0.137 | 0.084 | 0.089 | 0.0865 | 0.0505 |
| **44** | **E-3-6** | **0.757** | **0.746** | **0.7515** | **0.084** | **0.089** | **0.0865** | **0.665** |
| 45 | E-3-7 | 0.112 | 0.107 | 0.1095 | 0.084 | 0.089 | 0.0865 | 0.023 |
| 46 | E-3-8 | 0.312 | 0.318 | 0.315 | 0.084 | 0.089 | 0.0865 | 0.2285 |
| 47 | E-3-9 | 0.137 | 0.144 | 0.1405 | 0.084 | 0.089 | 0.0865 | 0.054 |
| 48 | E-3-10 | 0.13 | 0.129 | 0.1295 | 0.084 | 0.089 | 0.0865 | 0.043 |
| **Senangkhanikhom district** | | | | | | | | |
| **Sample Number** | **Sample ID** | **OD1** | **OD2** | **OD Average** | **Assay diluent 1** | **Assay diluent 2** | **Average** | **Analyzed** |
| 1 | F-1-1 | 0.087 | 0.093 | 0.09 | 0.084 | 0.089 | 0.0865 | 0.0035 |
| 2 | F-1-2 | 0.343 | 0.34 | 0.3415 | 0.084 | 0.089 | 0.0865 | 0.255 |
| 3 | F-1-3 | 0.229 | 0.23 | 0.2295 | 0.084 | 0.089 | 0.0865 | 0.143 |
| 4 | F-1-4 | 0.34 | 0.334 | 0.337 | 0.084 | 0.089 | 0.0865 | 0.2505 |
| 5 | F-1-5 | 0.151 | 0.147 | 0.149 | 0.084 | 0.089 | 0.0865 | 0.0625 |
| 6 | F-1-6 | 0.505 | 0.482 | 0.4935 | 0.084 | 0.089 | 0.0865 | 0.407 |
| 7 | F-1-7 | 0.236 | 0.256 | 0.246 | 0.084 | 0.089 | 0.0865 | 0.1595 |
| 8 | F-1-8 | 0.123 | 0.12 | 0.1215 | 0.084 | 0.089 | 0.0865 | 0.035 |
| 9 | F-2-1 | 0.213 | 0.201 | 0.207 | 0.084 | 0.089 | 0.0865 | 0.1205 |
| 10 | F-2-2 | 0.111 | 0.126 | 0.1185 | 0.084 | 0.089 | 0.0865 | 0.032 |
| 11 | F-2-3 | 0.197 | 0.217 | 0.207 | 0.084 | 0.089 | 0.0865 | 0.1205 |
| 12 | F-2-4 | 0.309 | 0.314 | 0.3115 | 0.084 | 0.089 | 0.0865 | 0.225 |
| 13 | F-2-5 | 0.177 | 0.188 | 0.1825 | 0.084 | 0.089 | 0.0865 | 0.096 |
| 14 | F-2-6 | 0.173 | 0.175 | 0.174 | 0.084 | 0.089 | 0.0865 | 0.0875 |
| **15** | **F-2-7** | **0.987** | **0.922** | **0.9545** | **0.084** | **0.089** | **0.0865** | **0.868** |
| **16** | **F-2-8** | **1.004** | **0.972** | **0.988** | **0.066** | **0.064** | **0.065** | **0.923** |
| 17 | F-2-9 | 0.139 | 0.138 | 0.1385 | 0.066 | 0.064 | 0.065 | 0.0735 |
| 18 | F-2-10 | 0.09 | 0.089 | 0.0895 | 0.066 | 0.064 | 0.065 | 0.0245 |
| 19 | F-2-11 | 0.082 | 0.08 | 0.081 | 0.066 | 0.064 | 0.065 | 0.016 |
| 20 | F-2-12 | 0.084 | 0.085 | 0.0845 | 0.066 | 0.064 | 0.065 | 0.0195 |
| 21 | F-2-13 | 0.144 | 0.143 | 0.1435 | 0.066 | 0.064 | 0.065 | 0.0785 |
| 22 | F-2-14 | 0.087 | 0.09 | 0.0885 | 0.066 | 0.064 | 0.065 | 0.0235 |
| 23 | F-2-15 | 0.171 | 0.153 | 0.162 | 0.066 | 0.064 | 0.065 | 0.097 |
| 24 | F-2-16 | 0.1 | 0.096 | 0.098 | 0.066 | 0.064 | 0.065 | 0.033 |
| 25 | F-2-17 | 0.428 | 0.387 | 0.4075 | 0.066 | 0.064 | 0.065 | 0.3425 |
| 26 | F-2-18 | 0.303 | 0.288 | 0.2955 | 0.066 | 0.064 | 0.065 | 0.2305 |
| 27 | F-2-19 | 0.086 | 0.094 | 0.09 | 0.066 | 0.064 | 0.065 | 0.025 |
| 28 | F-2-20 | 0.083 | 0.084 | 0.0835 | 0.066 | 0.064 | 0.065 | 0.0185 |
| 29 | F-2-21 | 0.119 | 0.122 | 0.1205 | 0.066 | 0.064 | 0.065 | 0.0555 |
| 30 | F-2-22 | 0.217 | 0.198 | 0.2075 | 0.066 | 0.064 | 0.065 | 0.1425 |
| **31** | **F-2-23** | **1.015** | **1.095** | **1.055** | **0.066** | **0.064** | **0.065** | **0.99** |
| 32 | F-2-24 | 0.123 | 0.124 | 0.1235 | 0.066 | 0.064 | 0.065 | 0.0585 |
| 33 | F-2-25 | 0.131 | 0.126 | 0.1285 | 0.066 | 0.064 | 0.065 | 0.0635 |
| 34 | F-2-26 | 0.098 | 0.096 | 0.097 | 0.066 | 0.064 | 0.065 | 0.032 |
| 35 | F-2-27 | 0.083 | 0.081 | 0.082 | 0.066 | 0.064 | 0.065 | 0.017 |
| 36 | F-2-28 | 0.118 | 0.118 | 0.118 | 0.066 | 0.064 | 0.065 | 0.053 |
| 37 | F-2-29 | 0.091 | 0.09 | 0.0905 | 0.066 | 0.064 | 0.065 | 0.0255 |
| 38 | F-2-30 | 0.079 | 0.077 | 0.078 | 0.066 | 0.064 | 0.065 | 0.013 |
| **39** | **F-3-1** | **0.84** | **0.876** | **0.858** | **0.066** | **0.064** | **0.065** | **0.793** |
| 40 | F-3-2 | 0.521 | 0.459 | 0.49 | 0.066 | 0.064 | 0.065 | 0.425 |
| 41 | F-3-3 | 0.098 | 0.095 | 0.0965 | 0.066 | 0.064 | 0.065 | 0.0315 |
| 42 | F-3-4 | 0.131 | 0.126 | 0.1285 | 0.066 | 0.064 | 0.065 | 0.0635 |
| 43 | F-3-5 | 0.339 | 0.328 | 0.3335 | 0.066 | 0.064 | 0.065 | 0.2685 |
| 44 | F-3-6 | 0.285 | 0.295 | 0.29 | 0.066 | 0.064 | 0.065 | 0.225 |
| 45 | F-3-7 | 0.298 | 0.312 | 0.305 | 0.066 | 0.064 | 0.065 | 0.24 |
| 46 | F-3-8 | 0.12 | 0.123 | 0.1215 | 0.066 | 0.064 | 0.065 | 0.0565 |
| 47 | F-3-9 | 0.143 | 0.144 | 0.1435 | 0.066 | 0.064 | 0.065 | 0.0785 |
| 48 | F-3-10 | 0.1 | 0.104 | 0.102 | 0.066 | 0.064 | 0.065 | 0.037 |
| **Chanuman district** | | | | | | | | |
| **Sample Number** | **Sample ID** | **OD1** | **OD2** | **OD Average** | **Assay diluent 1** | **Assay diluent 2** | **Average** | **Analyzed** |
| 1 | G-1-1 | 0.117 | 0.112 | 0.1145 | 0.066 | 0.064 | 0.065 | 0.0495 |
| 2 | G-1-2 | 0.13 | 0.132 | 0.131 | 0.066 | 0.064 | 0.065 | 0.066 |
| 3 | G-1-3 | 0.096 | 0.095 | 0.0955 | 0.066 | 0.064 | 0.065 | 0.0305 |
| 4 | G-1-4 | 0.265 | 0.239 | 0.252 | 0.066 | 0.064 | 0.065 | 0.187 |
| 5 | G-1-5 | 0.109 | 0.101 | 0.105 | 0.066 | 0.064 | 0.065 | 0.04 |
| 6 | G-1-6 | 0.192 | 0.186 | 0.189 | 0.066 | 0.064 | 0.065 | 0.124 |
| 7 | G-1-7 | 0.244 | 0.245 | 0.2445 | 0.066 | 0.064 | 0.065 | 0.1795 |
| 8 | G-2-1 | 0.093 | 0.104 | 0.0985 | 0.066 | 0.064 | 0.065 | 0.0335 |
| 9 | G-2-2 | 0.093 | 0.097 | 0.095 | 0.066 | 0.064 | 0.065 | 0.03 |
| 10 | G-2-3 | 0.18 | 0.183 | 0.1815 | 0.066 | 0.064 | 0.065 | 0.1165 |
| 11 | G-2-4 | 0.09 | 0.091 | 0.0905 | 0.066 | 0.064 | 0.065 | 0.0255 |
| 12 | G-2-5 | 0.154 | 0.147 | 0.1505 | 0.066 | 0.064 | 0.065 | 0.0855 |
| 13 | G-2-6 | 0.122 | 0.125 | 0.1235 | 0.066 | 0.064 | 0.065 | 0.0585 |
| 14 | G-2-7 | 0.081 | 0.082 | 0.0815 | 0.066 | 0.064 | 0.065 | 0.0165 |
| 15 | G-2-8 | 0.179 | 0.538 | 0.3585 | 0.066 | 0.064 | 0.065 | 0.2935 |
| **16** | **G-2-9** | **2.836** | **2.895** | **2.8655** | **0.066** | **0.064** | **0.065** | **2.8005** |
| 17 | G-2-10 | 0.397 | 0.357 | 0.377 | 0.066 | 0.064 | 0.065 | 0.312 |
| 18 | G-2-11 | 0.205 | 0.2 | 0.2025 | 0.066 | 0.064 | 0.065 | 0.1375 |
| 19 | G-2-12 | 0.08 | 0.083 | 0.0815 | 0.066 | 0.064 | 0.065 | 0.0165 |
| 20 | G-2-13 | 0.237 | 0.217 | 0.227 | 0.066 | 0.064 | 0.065 | 0.162 |
| 21 | G-2-14 | 0.577 | 0.531 | 0.554 | 0.066 | 0.064 | 0.065 | 0.489 |
| 22 | G-2-15 | 0.115 | 0.111 | 0.113 | 0.066 | 0.064 | 0.065 | 0.048 |
| 23 | G-2-16 | 0.123 | 0.122 | 0.1225 | 0.066 | 0.064 | 0.065 | 0.0575 |
| 24 | G-2-17 | 0.084 | 0.09 | 0.087 | 0.066 | 0.064 | 0.065 | 0.022 |
| 25 | G-2-18 | 0.089 | 0.089 | 0.089 | 0.066 | 0.064 | 0.065 | 0.024 |
| 26 | G-2-19 | 0.183 | 0.174 | 0.1785 | 0.066 | 0.064 | 0.065 | 0.1135 |
| 27 | G-2-20 | 0.088 | 0.088 | 0.088 | 0.066 | 0.064 | 0.065 | 0.023 |
| 28 | G-2-21 | 0.148 | 0.139 | 0.1435 | 0.066 | 0.064 | 0.065 | 0.0785 |
| 29 | G-2-22 | 0.314 | 0.303 | 0.3085 | 0.066 | 0.064 | 0.065 | 0.2435 |
| 30 | G-2-23 | 0.426 | 0.407 | 0.4165 | 0.066 | 0.064 | 0.065 | 0.3515 |
| 31 | G-2-24 | 0.074 | 0.072 | 0.073 | 0.066 | 0.064 | 0.065 | 0.008 |
| 32 | G-2-25 | 0.115 | 0.113 | 0.114 | 0.066 | 0.064 | 0.065 | 0.049 |
| 33 | G-2-26 | 0.087 | 0.085 | 0.086 | 0.066 | 0.064 | 0.065 | 0.021 |
| 34 | G-2-27 | 0.084 | 0.084 | 0.084 | 0.066 | 0.064 | 0.065 | 0.019 |
| 35 | G-2-28 | 0.118 | 0.112 | 0.115 | 0.066 | 0.064 | 0.065 | 0.05 |
| 36 | G-3-1 | 0.112 | 0.11 | 0.111 | 0.066 | 0.064 | 0.065 | 0.046 |
| 37 | G-3-2 | 0.475 | 0.467 | 0.471 | 0.066 | 0.064 | 0.065 | 0.406 |
| 38 | G-3-3 | 0.16 | 0.157 | 0.1585 | 0.066 | 0.064 | 0.065 | 0.0935 |
| 39 | G-3-4 | 0.367 | 0.362 | 0.3645 | 0.066 | 0.064 | 0.065 | 0.2995 |
| 40 | G-3-5 | 0.191 | 0.193 | 0.192 | 0.066 | 0.064 | 0.065 | 0.127 |
| 41 | G-3-6 | 0.494 | 0.505 | 0.4995 | 0.066 | 0.064 | 0.065 | 0.4345 |
| 42 | G-3-7 | 0.183 | 0.182 | 0.1825 | 0.066 | 0.064 | 0.065 | 0.1175 |
| 43 | G-3-8 | 0.107 | 0.111 | 0.109 | 0.066 | 0.064 | 0.065 | 0.044 |
| **44** | **G-3-9** | **1.323** | **1.251** | **1.287** | **0.066** | **0.064** | **0.065** | **1.222** |
