# Supplementary material for: Evaluation of culture- and PCR-based methods for detecting Burkholderia pseudomallei in soil samples in Thailand
Source: PLoS Negl Trop Dis. 2026 Jan 2;20(1):e0013840. doi: 10.1371/journal.pntd.0013840 (PMC12758721; doi:10.1371/journal.pntd.0013840)
Supplement: S7 Table — The table presents the detection results obtained from direct culture on Ashdown agar and enrichment culture in ACER broth at 5 and 9 days of incubation. (DOCX) [file pntd.0013840.s008.docx]

**S7 Table. Detection of *B. pseudomallei* in soil samples using culture methods.** The table presents the detection results obtained from direct culture on Ashdown agar and enrichment culture in ACER broth at 5 and 9 days of incubation.

| **Mueang Amnat Charoen** | | | | |
| --- | --- | --- | --- | --- |
| **Participant code** | **Soil sample ID** | **Result of *B. pseudomallei* culture** | | |
|  |  | **Direct plating on Ashdown** | **Enrichment culture on ACER (D5)** | **Enrichment culture on ACER (D9)** |
| A-1-9 | M-1-S01 | Negative | Negative | Negative |
| A-1-9 | M-1-S02 | Negative | Negative | Negative |
| A-1-9 | M-1-S03 | Negative | Negative | Negative |
| A-1-9 | M-1-S04 | Negative | Negative | Negative |
| A-1-9 | M-1-S05 | Negative | Negative | Negative |
| A-1-9 | M-1-S06 | Negative | Negative | Negative |
| A-1-9 | M-1-S07 | Negative | Negative | Negative |
| A-1-9 | M-1-S08 | Negative | Negative | Negative |
| A-1-9 | M-1-S09 | Negative | Negative | Negative |
| A-1-9 | M-1-S10 | Negative | Negative | Negative |
| A-1-9 | M-1-S11 | Negative | Negative | Negative |
| A-1-9 | M-1-S12 | Negative | Negative | Negative |
| A-1-9 | M-1-S13 | Negative | Negative | Negative |
| A-1-9 | M-1-S14 | Negative | Negative | Negative |
| A-1-9 | M-1-S15 | Negative | Negative | Negative |
| A-3-27 | M-2-S01 | Negative | Negative | Negative |
| A-3-27 | M-2-S02 | Negative | Negative | Negative |
| A-3-27 | M-2-S03 | Negative | Negative | Negative |
| A-3-27 | M-2-S04 | Negative | Negative | Negative |
| A-3-27 | M-2-S05 | Negative | Negative | Negative |
| A-3-27 | M-2-S06 | Negative | Negative | Negative |
| A-3-27 | M-2-S07 | Negative | Negative | Negative |
| A-3-27 | M-2-S08 | Negative | Negative | Negative |
| A-3-27 | M-2-S09 | Negative | Negative | Negative |
| A-3-27 | M-2-S10 | Negative | Negative | Negative |
| A-3-27 | M-2-S11 | Negative | Negative | Negative |
| A-3-27 | M-2-S12 | Negative | Negative | Negative |
| A-3-27 | M-2-S13 | Negative | Negative | Negative |
| A-3-27 | M-2-S14 | Negative | Negative | Negative |
| A-3-27 | M-2-S15 | Negative | Negative | Negative |
| A-3-27 | M-2-S16 | Negative | Negative | Negative |
| A-3-27 | M-2-S17 | Negative | Negative | Negative |
| A-3-27 | M-2-S18 | Negative | Negative | Negative |
| A-3-27 | M-2-S19 | Negative | Negative | Negative |
| A-3-27 | M-2-S20 | Negative | Negative | Negative |
| A-1-16 | M-3-S01 | Negative | Negative | Negative |
| A-1-16 | M-3-S02 | Negative | Negative | Negative |
| A-1-16 | M-3-S03 | Negative | Negative | **Positive** |
| A-1-16 | M-3-S04 | Negative | Negative | Negative |
| A-1-16 | M-3-S05 | Negative | Negative | Negative |
| A-1-16 | M-3-S06 | Negative | Negative | Negative |
| A-1-16 | M-3-S07 | Negative | Negative | Negative |
| A-1-16 | M-3-S08 | **Positive** | Negative | **Positive** |
| A-1-16 | M-3-S09 | **Positive** | **Positive** | **Positive** |
| A-1-16 | M-3-S10 | Negative | Negative | Negative |
| A-1-16 | M-3-S11 | Negative | Negative | Negative |
| A-1-16 | M-3-S12 | Negative | Negative | Negative |
| A-1-16 | M-3-S13 | Negative | Negative | Negative |
| A-1-16 | M-3-S14 | Negative | Negative | Negative |
| A-1-16 | M-3-S15 | Negative | Negative | Negative |
| A-1-16 | M-3-S16 | Negative | Negative | Negative |
| A-1-16 | M-3-S17 | **Positive** | Negative | Negative |
| A-1-16 | M-3-S18 | Negative | Negative | **Positive** |
| A-1-16 | M-3-S19 | Negative | Negative | Negative |
| A-1-16 | M-3-S20 | Negative | Negative | Negative |
| A-1-16 | M-3-S21 | Negative | Negative | Negative |
| A-1-16 | M-3-S22 | Negative | Negative | Negative |
| A-1-16 | M-3-S23 | Negative | Negative | Negative |
| A-1-16 | M-3-S24 | Negative | Negative | Negative |
| A-1-16 | M-3-S25 | Negative | Negative | Negative |
| A-2-17 | M-4-S01 | Negative | Negative | Negative |
| A-2-17 | M-4-S02 | Negative | Negative | Negative |
| A-2-17 | M-4-S03 | Negative | Negative | Negative |
| A-2-17 | M-4-S04 | Negative | **Positive** | Negative |
| A-2-17 | M-4-S05 | Negative | **Positive** | Negative |
| A-2-17 | M-4-S07 | Negative | Negative | Negative |
| A-2-17 | M-4-S08 | Negative | Negative | Negative |
| A-2-17 | M-4-S09 | Negative | Negative | Negative |
| A-2-17 | M-4-S10 | Negative | **Positive** | **Positive** |
| A-2-17 | M-4-S11 | Negative | **Positive** | **Positive** |
| A-2-17 | M-4-S12 | Negative | Negative | Negative |
| A-2-17 | M-4-S13 | Negative | Negative | Negative |
| A-2-17 | M-4-S14 | Negative | Negative | Negative |
| A-2-17 | M-4-S15 | Negative | Negative | Negative |
| A-2-17 | M-4-S16 | Negative | Negative | Negative |
| A-2-17 | M-4-S17 | Negative | Negative | Negative |
| A-2-17 | M-4-S18 | Negative | Negative | Negative |
| A-2-17 | M-4-S19 | Negative | Negative | Negative |
| A-2-17 | M-4-S20 | Negative | Negative | Negative |
| A-2-48 | M-5-S01 | Negative | Negative | Negative |
| A-2-48 | M-5-S02 | Negative | Negative | Negative |
| A-2-48 | M-5-S03 | Negative | Negative | Negative |
| A-2-48 | M-5-S04 | Negative | Negative | Negative |
| A-2-48 | M-5-S05 | Negative | Negative | Negative |
| A-2-48 | M-5-S06 | Negative | Negative | Negative |
| A-2-48 | M-5-S07 | Negative | Negative | Negative |
| A-2-48 | M-5-S08 | Negative | Negative | **Positive** |
| A-2-48 | M-5-S09 | Negative | Negative | Negative |
| A-2-48 | M-5-S10 | Negative | Negative | Negative |
| A-2-48 | M-5-S11 | Negative | Negative | Negative |
| A-2-48 | M-5-S12 | Negative | Negative | Negative |
| A-2-48 | M-5-S13 | Negative | Negative | Negative |
| A-2-48 | M-5-S14 | Negative | Negative | Negative |
| A-2-48 | M-5-S15 | Negative | Negative | Negative |
| A-2-48 | M-5-S16 | Negative | Negative | Negative |
| A-2-48 | M-5-S17 | Negative | Negative | Negative |
| A-2-48 | M-5-S18 | Negative | Negative | Negative |
| A-2-48 | M-5-S19 | Negative | **Positive** | **Positive** |
| A-2-48 | M-5-S20 | Negative | Negative | Negative |
| A-2-48 | M-5-S21 | Negative | Negative | Negative |
| A-2-48 | M-5-S22 | Negative | Negative | Negative |
| A-2-48 | M-5-S23 | Negative | Negative | Negative |
| A-2-48 | M-5-S25 | Negative | Negative | Negative |
| A-2-48 | M-5-S26 | Negative | Negative | Negative |
| A-2-48 | M-5-S27 | Negative | Negative | Negative |
| A-2-48 | M-5-S28 | Negative | Negative | Negative |
| A-2-48 | M-5-S29 | Negative | Negative | Negative |
| A-2-48 | M-5-S30 | Negative | Negative | Negative |
| **Pathum Ratchawongsa** | | | | |
| **Participant code** | **SampleID** | **Result on Ashdown** | **Result on ACER (D5)** | **Result on ACER (D9)** |
| E-2-15 | P-1-S01 | Negative | Negative | Negative |
| E-2-15 | P-1-S02 | Negative | Negative | Negative |
| E-2-15 | P-1-S03 | Negative | Negative | Negative |
| E-2-15 | P-1-S04 | Negative | **Positive** | Negative |
| E-2-15 | P-1-S05 | Negative | Negative | Negative |
| E-2-15 | P-1-S06 | Negative | Negative | Negative |
| E-2-15 | P-1-S07 | Negative | Negative | Negative |
| E-2-15 | P-1-S08 | Negative | Negative | Negative |
| E-2-15 | P-1-S09 | Negative | Negative | Negative |
| E-2-15 | P-1-S10 | **Positive** | **Positive** | **Positive** |
| E-2-15 | P-1-S11 | Negative | Negative | Negative |
| E-2-15 | P-1-S12 | Negative | Negative | Negative |
| E-2-15 | P-1-S13 | Negative | Negative | Negative |
| E-2-15 | P-1-S14 | Negative | Negative | Negative |
| E-2-15 | P-1-S15 | Negative | Negative | Negative |
| E-2-15 | P-1-S16 | Negative | Negative | Negative |
| E-2-15 | P-1-S17 | Negative | Negative | Negative |
| E-2-15 | P-1-S18 | Negative | Negative | Negative |
| E-2-15 | P-1-S19 | Negative | **Positive** | Negative |
| E-2-15 | P-1-S20 | Negative | Negative | Negative |
| E-2-15 | P-1-S21 | **Positive** | Negative | Negative |
| E-2-15 | P-1-S22 | Negative | Negative | Negative |
| E-2-15 | P-1-S23 | Negative | Negative | **Positive** |
| E-2-15 | P-1-S24 | **Positive** | **Positive** | **Positive** |
| E-2-15 | P-1-S25 | Negative | Negative | Negative |
| E-2-15 | P-1-S26 | Negative | Negative | Negative |
| E-2-15 | P-1-S27 | Negative | Negative | Negative |
| E-2-15 | P-1-S28 | Negative | Negative | **Positive** |
| E-2-15 | P-1-S29 | Negative | Negative | Negative |
| E-2-15 | P-1-S30 | **Positive** | **Positive** | **Positive** |
| E-2-30 | P-2-S01 | Negative | Negative | Negative |
| E-2-30 | P-2-S02 | Negative | Negative | Negative |
| E-2-30 | P-2-S03 | Negative | Negative | Negative |
| E-2-30 | P-2-S04 | Negative | Negative | Negative |
| E-2-30 | P-2-S05 | Negative | Negative | Negative |
| E-2-30 | P-2-S06 | Negative | Negative | Negative |
| E-2-30 | P-2-S07 | Negative | Negative | Negative |
| E-2-30 | P-2-S08 | Negative | Negative | Negative |
| E-2-30 | P-2-S09 | Negative | Negative | Negative |
| E-2-30 | P-2-S10 | Negative | Negative | Negative |
| E-2-30 | P-2-S11 | Negative | Negative | Negative |
| E-2-30 | P-2-S12 | Negative | Negative | Negative |
| E-2-30 | P-2-S13 | Negative | Negative | Negative |
| E-2-30 | P-2-S14 | Negative | Negative | Negative |
| E-2-30 | P-2-S15 | Negative | Negative | Negative |
| E-1-1 | P-3-S01 | Negative | Negative | Negative |
| E-1-1 | P-3-S02 | Negative | Negative | Negative |
| E-1-1 | P-3-S03 | Negative | Negative | Negative |
| E-1-1 | P-3-S04 | Negative | Negative | Negative |
| E-1-1 | P-3-S05 | Negative | Negative | Negative |
| E-1-1 | P-3-S06 | Negative | Negative | Negative |
| E-1-1 | P-3-S07 | Negative | Negative | Negative |
| E-1-1 | P-3-S08 | Negative | Negative | Negative |
| E-1-1 | P-3-S09 | Negative | Negative | Negative |
| E-1-1 | P-3-S10 | Negative | Negative | Negative |
| E-1-1 | P-3-S11 | Negative | Negative | Negative |
| E-1-1 | P-3-S12 | Negative | Negative | Negative |
| E-1-1 | P-3-S13 | Negative | Negative | Negative |
| E-1-1 | P-3-S14 | Negative | Negative | Negative |
| E-1-1 | P-3-S15 | Negative | Negative | Negative |
| **Chanuman** | | | | |
| **Participant code** | **SampleID** | **Result on Ashdown** | **Result on ACER (D5)** | **Result on ACER (D9)** |
| G-2-9 | C-1-S01 | Negative | Negative | Negative |
| G-2-9 | C-1-S02 | Negative | Negative | Negative |
| G-2-9 | C-1-S03 | Negative | Negative | Negative |
| G-2-9 | C-1-S04 | Negative | Negative | Negative |
| G-2-9 | C-1-S05 | Negative | Negative | Negative |
| G-2-9 | C-1-S06 | Negative | Negative | Negative |
| G-2-9 | C-1-S07 | Negative | Negative | Negative |
| G-2-9 | C-1-S08 | Negative | Negative | Negative |
| G-2-9 | C-1-S09 | Negative | Negative | Negative |
| G-2-9 | C-1-S10 | Negative | Negative | Negative |
| G-2-9 | C-1-S11 | **Positive** | **Positive** | **Positive** |
| G-2-9 | C-1-S12 | Negative | Negative | Negative |
| G-2-9 | C-1-S13 | Negative | Negative | Negative |
| G-2-9 | C-1-S14 | Negative | Negative | Negative |
| G-2-9 | C-1-S15 | Negative | Negative | Negative |
| G-3-9 | C-2-S01 | Negative | Negative | Negative |
| G-3-9 | C-2-S02 | Negative | Negative | Negative |
| G-3-9 | C-2-S03 | Negative | Negative | Negative |
| G-3-9 | C-2-S04 | Negative | Negative | Negative |
| G-3-9 | C-2-S05 | Negative | Negative | Negative |
| G-3-9 | C-2-S06 | Negative | Negative | Negative |
| G-3-9 | C-2-S07 | Negative | Negative | Negative |
| G-3-9 | C-2-S08 | Negative | Negative | Negative |
| G-3-9 | C-2-S09 | Negative | Negative | Negative |
| G-2-7 | C-3-S01 | Negative | Negative | Negative |
| G-2-7 | C-3-S02 | Negative | Negative | Negative |
| G-2-7 | C-3-S03 | Negative | Negative | Negative |
| G-2-7 | C-3-S04 | Negative | Negative | Negative |
| G-2-7 | C-3-S05 | Negative | Negative | Negative |
| G-2-7 | C-3-S06 | Negative | Negative | Negative |
| G-2-7 | C-3-S07 | Negative | Negative | Negative |
| G-2-7 | C-3-S08 | Negative | Negative | Negative |
| G-2-7 | C-3-S09 | Negative | Negative | Negative |
| G-2-7 | C-3-S10 | Negative | Negative | Negative |
| G-2-7 | C-3-S11 | Negative | Negative | Negative |
| G-2-7 | C-3-S12 | Negative | Negative | Negative |
| G-2-7 | C-3-S13 | Negative | Negative | Negative |
| G-2-7 | C-3-S14 | Negative | Negative | Negative |
| G-2-7 | C-3-S15 | Negative | Negative | Negative |
| G-2-7 | C-3-S16 | Negative | Negative | Negative |
| G-2-7 | C-3-S17 | Negative | Negative | Negative |
| G-2-7 | C-3-S18 | Negative | Negative | Negative |
| G-2-7 | C-3-S19 | Negative | Negative | Negative |
| G-2-7 | C-3-S20 | Negative | Negative | Negative |
| G-2-7 | C-3-S21 | Negative | Negative | Negative |
| G-2-7 | C-3-S22 | Negative | Negative | Negative |
| G-2-7 | C-3-S23 | Negative | Negative | Negative |
| G-2-7 | C-3-S24 | Negative | Negative | Negative |
| G-2-7 | C-3-S25 | Negative | Negative | Negative |
| G-2-7 | C-3-S26 | Negative | Negative | Negative |
| G-2-7 | C-3-S27 | Negative | Negative | Negative |
| G-2-7 | C-3-S28 | Negative | Negative | Negative |
| G-2-7 | C-3-S29 | Negative | Negative | Negative |
| G-2-7 | C-3-S30 | Negative | Negative | Negative |
| G-2-24 | C-4-S01 | Negative | Negative | Negative |
| G-2-24 | C-4-S02 | Negative | Negative | Negative |
| G-2-24 | C-4-S03 | Negative | Negative | Negative |
| G-2-24 | C-4-S04 | Negative | Negative | Negative |
| G-2-24 | C-4-S05 | Negative | Negative | Negative |
| G-2-24 | C-4-S06 | Negative | Negative | Negative |
| G-2-24 | C-4-S07 | Negative | Negative | Negative |
| G-2-24 | C-4-S08 | Negative | Negative | Negative |
| G-2-24 | C-4-S09 | Negative | Negative | Negative |
| G-2-24 | C-4-S10 | Negative | Negative | Negative |
| G-2-24 | C-4-S11 | Negative | Negative | Negative |
| G-2-24 | C-4-S12 | Negative | Negative | Negative |
| G-2-24 | C-4-S13 | Negative | Negative | Negative |
| G-2-24 | C-4-S14 | Negative | Negative | Negative |
| G-2-24 | C-4-S15 | Negative | Negative | Negative |

*Negative -*B. pseudomallei* negative soil samples

*Positive -*B. pseuodmallei* positive soil samples
