# Supplementary material for: Evaluation of culture- and PCR-based methods for detecting Burkholderia pseudomallei in soil samples in Thailand
Source: PLoS Negl Trop Dis. 2026 Jan 2;20(1):e0013840. doi: 10.1371/journal.pntd.0013840 (PMC12758721; doi:10.1371/journal.pntd.0013840)
Supplement: S8 Table — Red color indicates samples that tested positive by each corresponding method. (DOCX) [file pntd.0013840.s009.docx]

**S8 Table.** **Summary of *B. pseudomallei*-positive soil samples detected by culture**

**and PCR methods in Amnat Charoen province.** Red color indicates samples that

tested positive by each corresponding method.

| **Sample ID** | **Districts** | **Positive samples** | | | | |
| --- | --- | --- | --- | --- | --- | --- |
|  |  | **Direct plating on Ashdown agar** | **ACER broth at day 5** | **ACER broth at day 9** | ***BPSS1187*-**  **PCR** | ***TTS1-***  ***orf2*-**  **PCR** |
| M-3-S03 | Mueang Amnat Charoen |  |  |  |  |  |
| M-3-S08 | Mueang Amnat Charoen |  |  |  |  |  |
| M-3-S09 | Mueang Amnat Charoen |  |  |  |  |  |
| M-3-S17 | Mueang Amnat Charoen |  |  |  |  |  |
| M-3-S18 | Mueang Amnat Charoen |  |  |  |  |  |
| M-4-S04 | Mueang Amnat Charoen |  |  |  |  |  |
| M-4-S05 | Mueang Amnat Charoen |  |  |  |  |  |
| M-4-S10 | Mueang Amnat Charoen |  |  |  |  |  |
| M-4-S11 | Mueang Amnat Charoen |  |  |  |  |  |
| M-5-S08 | Mueang Amnat Charoen |  |  |  |  |  |
| M-5-S19 | Mueang Amnat Charoen |  |  |  |  |  |
| P-1-S04 | Pathum Ratchawongsa |  |  |  |  |  |
| P-1-S10 | Pathum Ratchawongsa |  |  |  |  |  |
| P-1-S19 | Pathum Ratchawongsa |  |  |  |  |  |
| P-1-S21 | Pathum Ratchawongsa |  |  |  |  |  |
| P-1-S23 | Pathum Ratchawongsa |  |  |  |  |  |
| P-1-S24 | Pathum Ratchawongsa |  |  |  |  |  |
| P-1-S28 | Pathum Ratchawongsa |  |  |  |  |  |
| P-1-S30 | Pathum Ratchawongsa |  |  |  |  |  |
| C-1-S11 | Chanuman |  |  |  |  |  |
| **Total** |  | **8/20** | **12/20** | **14/20** | **2/20** | **2/20** |
