# Supplementary material for: Evaluation of culture- and PCR-based methods for detecting Burkholderia pseudomallei in soil samples in Thailand
Source: PLoS Negl Trop Dis. 2026 Jan 2;20(1):e0013840. doi: 10.1371/journal.pntd.0013840 (PMC12758721; doi:10.1371/journal.pntd.0013840)
Supplement: Attachment — Submitted filename: Suttisak et al review.docx [file pntd.0013840.s010.docx]

**Suttisak et al review**

**Summary of the research and your overall impression**

The study attempts to reveal associations with Bps soil prevalence and sero-prevalence in a community recognised as melioidosis endemic, but less well described that the region is boarders, Ubon Ratchathani. In preparation for the soil survey an evaluation of different techniques and media used for environmental Bps isolation was undertaken.

As the authors state, it is critical that methods of Bps environmental detection be tested and optimised to support the global efforts in developing a consensus of how Bps prevalence in the environment is determined. So, work such as this is an important contribution to that cause.

The study compares different approaches of traditional culture-based techniques, with and without pre-enrichment, and then tests direct molecular detection of Bps, in a in a series of controlled experiments. In validating the molecular techniques, authors used culture positive samples as the denominator to test sensitivity of the direct molecular assays and found to reveal poor sensitivity.

Subsequent soil sampling and testing was guided by the sero-survey and the two culture techniques were used and compared, in field use. One direct plating and one enrichment method before direct plating. The molecular assay was not deployed in the field survey. However, 20 culture positive field samples were subjected to the molecular method which failed to detect 18 of the 20 culture confirmed samples.

**Major Issues**

Previous studies have determined the limitations of culture based environmental detection, used for epidemiological purposes, given its relatively poor sensitivity. This may explain the poor correlation between “soil prevalence” and sero-prevalence in this study.

The media optimisation experiments where conducted either with pure cultures from PBS or from Bps in sterile soil. Therefore, the inherent competition of the true environmental matrix and the selectively of the media could not be determined.

As the authors suggest, conducting the study in the wet season, when exposure to the organism is likely to be higher due increase environmental liberation, might produce different results. The disappointing molecular results have been appropriately discussed, however, as the authors mention, other investigators have published more successful results than this study could have perhaps benefited from is the experimental designed was informed by these studies. The study is well conducted, however, it is unclear if this work significantly describes an outcome that advances our knowledge of the use of molecular techniques used for environmental detection of Bps. Could the paper be re-addressed as a study into the associated of Bps environmental prevalence and sero-prevalence and have the assay validation as a material and method, rather than the main feature?

**Minor Issues**

**Line 69:** The authors reference the 2013 published consensus guidelines as justifying why culture is gold standard for environmental detection. The guide is being updated but importantly, may studies since then have been published demonstrating increased sensitivity of Bps environmental detection compared to culture for epidemiological purposes

**Line 83-100:** Indeed, perhaps addressing the rationale a little more clearly why the authors chose their method of molecular testing, given these recent reports, would be useful?

**Lines 109-110:** Environmental prevalence of an endemic community as guided by a seroreactivity of a sample of the population? Not the environmental prevalence of samples?

**Lines 141-152:** What do the authors mean about “efficiency” and what might be the hypothesis that drives this aspect of optimisation from pure cultures subbed directly from stock? Morphology and successful cultivation from stock as determined by numbers?

**Line 164:** please explain why soil was autoclaved. Clearly the relative selectivity of the broth media could not be tested in this design.

**Line 178:** so 10x 250mg of previously autoclaved soil each containing a unique strain of Bps?

**Lines 230-231**: Please rewrite sentence “seropositive households”?

**Line 246:** enrichment in ACER broth followed by culture of broth onto ASH? – **same as line 406**

**Lines 279-295:** Please explain why a relative colonial morphology and relative enumeration of Bps comparison within a bukholderiales population wasn’t also conducted. The may have produced different results as the agar systems may have had different selectivity properties.

**Lines 405 – 408**: Please discuss (in discussion) how/if the media optimisation experiments might not reflect true selectively and sensitivity when used in the field site

**Lines 464-470**: yes, from pure cultures but it would be interesting is how these media perform on mixed culture with Bps plus other Burkholderiales – please discuss this. Do you think it is a limitation of your study?

**Line 488:** yes, please discuss why this wasn’t also considered in the comparisons of ASH and Acer agars

**Line 501**: is there any evidence of how long IgG detected in assay lingers? Where the results published in UR from a study that was also conducted in the dry season? Perhaps this community, as the soil prevalence also perhaps suggest, just less endemic that UR?

**Lines 555-561**: explain why PCR wasn’t conducted on pre-enriched or culture samples given authors have cited literature that reports better sensitivity
