# Supplementary material for: Evaluation of culture- and PCR-based methods for detecting Burkholderia pseudomallei in soil samples in Thailand
Source: PLoS Negl Trop Dis. 2026 Jan 2;20(1):e0013840. doi: 10.1371/journal.pntd.0013840 (PMC12758721; doi:10.1371/journal.pntd.0013840)
Supplement: Attachment — Submitted filename: Point by point response for reviewer_Final.docx [file pntd.0013840.s012.docx]

**Evaluation of culture- and PCR-based methods for detecting *Burkholderia pseudomallei* in soil samples in Thailand**

**Reviewer Comments:**

**Reviewer 2**

**Comments:**

1. Presumably, the soil samples for DNA extraction and for the cultures were separate sub-samples of the original 200 g samples? Given that the distribution of *B. pseudomallei* can vary greatly even within a small volume, the large differences in the soil quantities used for the PCR and culture protocols could at least partly explain the low PCR positivity rate of culture-positive samples (?).

We appreciate the reviewer’s feedback. We have added the following text to the limitations section of the Discussion:

Lines 622-626: “In addition, the relatively small amount of soil used for DNA extraction (approximately 250 mg) may have further limited the sensitivity of real-time PCR, as *B. pseudomallei* is unevenly distributed in soil. Consequently, randomly selected sub-samples may not have contained sufficient bacterial cells for detection, thereby reducing assay sensitivity.”

2. Line 490 Reference not correct?

We thank the reviewer for pointing out this error. We have corrected the reference to accordingly.

Lines 519-521: “Additionally, erythritol, used as a carbon source in enrichment media, has been demonstrated to promote *B. pseudomallei* proliferation and improve recovery rates from environmental samples (Yabuuchi, Kosako et al. 1992, Assig, Lichtenegger et al. 2023).”

3. Line 512 to 513 Meaning 20% higher among younger people than the average?

We thank the reviewer for pointing this out. We realized the wording in the previous version of the manuscript was inaccurate. We have corrected the text to reflect the actual data reported by Cheng et al. (2006).

Lines 543-546: “This contrasts with previous findings from northeast Thailand, where high antibody titers were detected in approximately 20% of 2,214 children aged <15 years in Udon Thani and 11% of children in Ubon Ratchathani, as determined using the IHA.”

4. Line 556 to 561 The authors only refer to previous studies that detected *B. pseudomallei* DNA after culture enrichment. However, there are a number of studies which also applied PCR directly to soil extracts. These studies should be cited and briefly discussed.

We appreciate the reviewer’s comment. We have revised the Discussion to include additional references on direct soil DNA extraction (Kaestli, Mayo et al. 2007, Trung, Hetzer et al. 2011).

Lines 588-594: “In contrast, previous studies have successfully detected *B. pseudomallei* DNA from soil samples by conducting PCR after enrichment using TBSS-C50 based erythritol broth, or ACER broth (Trinh, Assig et al. 2019, Assig, Lichtenegger et al. 2023) or by extracting DNA from direct soil samples (Kaestli, Mayo et al. 2007, Trung, Hetzer et al. 2011). This discrepancy may reflect differences in DNA extraction efficiency, the effectiveness of inhibitor removal, soil composition, and the smaller quantities of soil used for DNA extraction, which may reduce the likelihood of detecting *B. pseudomallei* given its heterogeneous distribution in soil.”

5. Line 559 ...from soil ( ) Reference should be added.

We appreciate the reviewer’s comment. We have now added the appropriate reference to support this statement in the revised manuscript.

Lines 588-591: “In contrast, previous studies have successfully detected *B. pseudomallei* DNA from soil samples by conducting PCR after enrichment using TBSS-C50 based erythritol broth, or ACER broth (Trinh, Assig et al. 2019, Assig, Lichtenegger et al. 2023) or by extracting DNA from direct soil samples (Kaestli, Mayo et al. 2007, Trung, Hetzer et al. 2011).”

6. Abstract line 39 to 43 The culture results of samples from seronegative and seropositive households should be provided

We thank the reviewer’s comment. We have revised in the abstract

Lines 45-47: “Overall, *B. pseudomallei* was detected in 14 of 129 samples (10.9%) from households of seropositive individuals and in 6 of 108 samples (5.5%) from households of seronegative individuals (*p* = 0.165).”

**Major issues:**

Previous studies have determined the limitations of culture based environmental detection, used for epidemiological purposes, given its relatively poor sensitivity. This may explain the poor correlation between “soil prevalence” and sero-prevalence in this study. The media optimisation experiments were conducted either with pure cultures from PBS or from Bps in sterile soil. Therefore, the inherent competition of the true environmental matrix and the selectively of the media could not be determined. As the authors suggest, conducting the study in the wet season, when exposure to the organism is likely to be higher due increase environmental liberation, might produce different results. The disappointing molecular results have been appropriately discussed, however, as the authors mention, other investigators have published more successful results than this study could have perhaps benefited from is the experimental designed was informed by these studies. The study is well conducted, however, it is unclear if this work significantly describes an outcome that advances our knowledge of the use of molecular techniques used for environmental detection of Bps. Could the paper be re-addressed as a study into the associated of Bps environmental prevalence and sero-prevalence and have the assay validation as a material and method, rather than the main feature?

We thank the reviewer for these constructive comments. We reanalyzed the results using two cut-off values to define seropositivity. The first cut-off value calculated as mean OD_450nm_ plus 1.5 standard deviation (OD = 0.418), was used to identify individuals with probable exposure to *B. pseudomallei*. The second cut-off (OD 1.165), as previously described (Pumpuang, Dunachie et al. 2017) , was used to identify individuals with probable melioidosis (see revised Fig 6 and Table 1). No correlation between soil prevalence and sero-prevalence. We agree that culture-based environmental detection methods have inherent sensitivity limitations, which likely contributed to this lack of correlation. We have clarified this point in the revised Discussion (Lines 614-620) and acknowledged that media optimization using sterile soil may not fully replicate the microbial competition present in natural environment (Lines 614-620). This limitation has been stated, with the suggestion that future studies include testing in unsterile soils (Lines 614-620).

Regarding the molecular detection, we have added further detail comparing our approach with those reported by other groups and highlighted potential methodological differences that may explain the lower sensitivity achieved here (Lines 588-594). We have emphasized that our findings contribute to the broader understanding of the challenges in applying molecular detection for B. pseudomallei in complex environmental samples (Lines 627-629).

While we appreciate the suggestion to reframe the manuscript primarily as an epidemiological correlation study, our main intention was to evaluate detection methodologies in the context of their future use for environmental surveillance. We have therefore retained assay validation as a central component of the study, but we have modified the framing of the Discussion to make clearer how these methodological insights inform interpretation of the soil–sero-prevalence relationship (Lines:597-612). We believe this balance reflects both the strengths and the limitations of the current work.

Lines 614-620: “Future studies should include a broader range of soil-dwelling bacteria to better assess the selectivity and overall microbial diversity influenced by each culture condition. In addition, the media optimization experiments were performed with sterile soil, which may not accurately reflect the microbial competition present in natural environments. Future studies should utilize non-sterile soils during media optimization to better reflect natural ecological conditions.”

Lines 588-594: “In contrast, previous studies have successfully detected *B. pseudomallei* DNA from soil samples by conducting PCR after enrichment using TBSS-C50 based erythritol broth, or ACER broth (Trinh, Assig et al. 2019, Assig, Lichtenegger et al. 2023) or by extracting DNA from direct soil samples (Kaestli, Mayo et al. 2007, Trung, Hetzer et al. 2011). This discrepancy may reflect differences in DNA extraction efficiency, the effectiveness of inhibitor removal, soil composition, and the smaller quantities of soil used for DNA extraction, which may reduce the likelihood of detecting *B. pseudomallei* given its heterogeneous distribution in soil.”

Lines 627-629: “Future studies should consider using prior enrichment or soil pellet for DNA extraction and incorporate validated internal controls to enhance the accuracy and reliability of real-time PCR assays.”

Lines 597-612: “In this study, we investigated whether the presence of *B. pseudomallei* in household soil samples was associated with the individual seroprevalence. However, the results revealed no statistically significant association between environmental detection of *B. pseudomallei* and seroprevalence, and several factors may contribute to the absence of a strong correlation. Seroprevalence reflects cumulative or past exposure, whereas culture-based methods detect the current and often localized presence of *B. pseudomallei* in the environment. The intermittent distribution of *B. pseudomallei* (Pongmala, Pierret et al. 2022), combined with individual variation in exposure behavior, such as farming or gardening activities that involve contact with contaminated soil and water (Wuthiekanun, Chierakul et al. 2006, Selvam, Ganapathy et al. 2022), may contribute to this discrepancy. Furthermore, host immune responses to *B. pseudomallei* are highly variable and can influence the detectability of antibody responses, with some individuals mounting strong cell-mediated immunity in the absence of measurable antibodies (Govan and Ketheesan 2004). In addition, environmental sampling limitations, such as low sampling density or non-representative site selection may lead to underestimation of *B. pseudomallei* prevalence in soil (Limmathurotsakul, Wuthiekanun et al. 2010). These factors may contribute to the discordance between the environmental prevalence of *B. pseudomallei* and human seroprevalence.”

**Minor issues:**

1. Line 69: The authors reference the 2013 published consensus guidelines as justifying why culture is gold standard for environmental detection. The guide is being updated but importantly, may studies since then have been published demonstrating increased sensitivity of Bps environmental detection compared to culture for epidemiological purposes

We thank the reviewer’s comment. In the Introduction Lines 102-115, we clarified the role of real-time PCR methods that improved the sensitivity of *B. pseudomallei* detection in environmental samples. For example, In Lines 108-110: “Real-time PCR has consistently shown higher detection rates for *B. pseudomallei* in environmental samples compared to culture methods using TBSS-C50 enrichment broth”. However, culture remains gold standard for *B. pseudomallei* detection whether in clinical or environmental in endemic area due to low cost for identification and can detect viable bacteria.

2. Line 83-100: Indeed, perhaps addressing the rationale a little more clearly why the authors chose their method of molecular testing, given these recent reports, would be useful?

We thank the reviewer for this comment.

In the Introduction (Lines: 102-115), we clarified the rationale for using real-time PCR by highlighting its high sensitivity and specificity. For example, studies of *TTS1*-PCR assays have reported detection rates of approximately 76% in soil samples, while specificity studies demonstrated 100% specificity for *B. pseudomallei* in spiked samples. In addition, recent work evaluating the *BPSS1187* gene showed a high sensitivity of around 76% when applied to human blood samples. We chose to focus on real-time PCR in our study because only a limited number of investigations have evaluated real-time PCR targeting the *BPSS1187* gene in environmental samples, and we aimed to address this knowledge gap.

3. Lines 109-110: Environmental prevalence of an endemic community as guided by a seroreactivity of a sample of the population? Not the environmental prevalence of samples?

We thank the reviewer for this comment. We agree that our wording at Lines 109–110 may have caused confusion. We have corrected it accordingly.

Lines 124-127: “We then examined the environmental prevalence of *B. pseudomallei* in 238 soil samples collected in Amnat Charoen province. These included soil surrounding the houses of individuals that were strongly positive (N = 6) and negative (N = 6) for Hcp1-specific antibodies.”

4. Lines 141-152: What do the authors mean about “efficiency” and what might be the hypothesis that drives this aspect of optimisation from pure cultures subbed directly from stock? Morphology and successful cultivation from stock as determined by numbers?

The “efficiency” referred to the ability of the media to support the growth and recovery of *B. pseudomallei*, which is particularly relevant because this organism is often present at low abundance in environmental samples. Our working hypothesis was that media showing higher recovery from spiked 10 environmental strains of *B. pseudomallei* (pure culture) would be better candidates for subsequent testing with environmental samples.

We acknowledge, however, that the use of pure cultures represents a limitation of this optimization step, as it does not fully reflect real environmental conditions where soil-dwelling bacteria and other microorganisms coexist and may influence selectivity and growth (Discussion, Lines: 614-620).

Lines 614-620: “Future studies should include a broader range of soil-dwelling bacteria to better assess the selectivity and overall microbial diversity influenced by each culture condition. In addition, the media optimization experiments were performed with sterile soil, which may not accurately reflect the microbial competition present in natural environments. Future studies should utilize non-sterile soils during media optimization to better reflect natural ecological conditions.”

5. Line 164: please explain why soil was autoclaved. Clearly the relative selectivity of the broth media could not be tested in this design.

Soil was autoclaved prior to inoculation to eliminate background microorganisms. This approach enabled us to evaluate the recovery of *B. pseudomallei* under controlled conditions without interference from native soil microbiota. However, this study design did not allow assessment of the relative selectivity of the broth media in the presence of natural microbial communities. We have noted this as a limitation in the Discussion (Lines: 614-620) and emphasized that future studies should use non-autoclaved soil or spike soil-dwelling bacteria to better evaluate broth media selectivity under realistic environmental conditions.

Lines 614-620: “Future studies should include a broader range of soil-dwelling bacteria to better assess the selectivity and overall microbial diversity influenced by each culture condition. In addition, the media optimization experiments were performed with sterile soil, which may not accurately reflect the microbial competition present in natural environments. Future studies should utilize non-sterile soils during media optimization to better reflect natural ecological conditions.”

6. Line 178: so 10x 250mg of previously autoclaved soil each containing a unique strain of Bps?

We have corrected the sentence.

Lines 193-196: “For real-time PCR assays, genomic DNA was extracted from 200 µl of bacterial pellet obtained from 1 ml of *B. pseudomallei* K96243 culture in LB broth or 250 mg of autoclaved soil spiked with ten environmental *B. pseudomallei* strains using the PowerSoil Pro Kit (QIAGEN) following the manufacturer’s instructions.”

7. Lines 230-231: Please rewrite sentence “seropositive households”?

We thank the reviewer for the comment. We have revised the sentence:

Lines 247-251: “Sampling locations were selected based on seroprevalence data from healthy populations, targeting households of healthy individuals who were strongly seropositive to *B. pseudomallei* (N = 6)*,* as determined by Hcp1-specific ELISA, while households of seronegative individuals (N = 6) were randomly selected for soil sampling.”

8. Line 246: enrichment in ACER broth followed by culture of broth onto ASH? – same as line 406

We thank the reviewer for the comment.

Line 270-276: “Five grams of soil from each sample was homogenized in 10 ml of PBS, and the suspensions were left to settle overnight at room temperature. A 500 µl aliquot of supernatant was then inoculated into 10 ml of ACER broth and incubated statically with a loose cap at 40°C for 5 and 9 days (Assig, Lichtenegger et al. 2023). Following incubation, 100 µl of broth culture was 10-fold serial diluted, 100 µl of each dilution was plated onto Ashdown agar, and the plates were incubated at 40°C for 4 days. Bacterial colonies were counted and confirmed to be *B. pseudomallei* (Novak, Glass et al. 2006, Duval, Elrod et al. 2014)”

9. Lines 279-295: Please explain why a relative colonial morphology and relative enumeration of Bps comparison within a Bukholderiales population wasn’t also conducted. They may have produced different results as the agar systems may have had different selectivity properties.

We appreciate the reviewer’s comment. In this study, our primary aim was to compare the relative colonial morphology and enumeration of *B. pseudomallei* across different selective media. Therefore, we did not extend our analysis to include the relative colonial morphology and enumeration of other Burkholderiales population. We acknowledge that such an approach may have provided additional insights, as differences in agar selectivity could influence both colony appearance and recovery rates. We have acknowledged this in the Discussion (Lines: 614-620) as a limitation of our study and to highlight that future work incorporating comparative colonial morphology and enumeration within mixed Burkholderiales populations would be valuable to further evaluate media performance.

Lines 614-620: “Future studies should include a broader range of soil-dwelling bacteria to better assess the selectivity and overall microbial diversity influenced by each culture condition. In addition, the media optimization experiments were performed with sterile soil, which may not accurately reflect the microbial competition present in natural environments. Future studies should utilize non-sterile soils during media optimization to better reflect natural ecological conditions.”

10. Lines 405 – 408: Please discuss (in discussion) how/if the media optimisation experiments might not reflect true selectively and sensitivity when used in the field site

We thank the reviewer for this valuable comment.

In the Discussion (Lines 614-620), we noted that our media optimization experiments were conducted under controlled laboratory conditions using pure cultures of *B. pseudomallei.* However, in the field, soil harbors diverse microbial communities and organic matter that can influence both the selectivity and sensitivity of culture-based detection. Competing organisms, indigenous soil bacteria, and potential inhibitory substances may reduce the recovery of *B. pseudomallei* in ways not observed under laboratory conditions. As a result, media optimization performed in the laboratory likely overestimates both sensitivity and selectivity. Therefore, the performance described here may not fully represent the true selectivity and sensitivity of these media in field applications. Future studies incorporating non-sterile soil or naturally contaminated environmental samples will be essential to validate the applicability of these optimized methods under realistic field conditions.

Lines 614-620: “Future studies should include a broader range of soil-dwelling bacteria to better assess the selectivity and overall microbial diversity influenced by each culture condition. In addition, the media optimization experiments were performed with sterile soil, which may not accurately reflect the microbial competition present in natural environments. Future studies should utilize non-sterile soils during media optimization to better reflect natural ecological conditions.”

11. Lines 464-470: yes, from pure cultures but it would be interesting is how these media perform on mixed culture with Bps plus other Burkholderiales – please discuss this. Do you think it is a limitation of your study?

We thank the reviewer for this important point. Our study was conducted using pure cultures of *B. pseudomallei* to evaluate the performance of the media under controlled conditions. We agree that testing with mixed cultures containing *B. pseudomallei*, other Burkholderiales species, and soil-dwelling bacteria would be valuable to assess assay performance under more realistic environmental conditions. We have acknowledged this in the discussion (Lines 614-620) as a limitation of our study and emphasized that further evaluation using mixed environmental cultures would provide a better reflection of field conditions.

Lines 614-620: “Future studies should include a broader range of soil-dwelling bacteria to better assess the selectivity and overall microbial diversity influenced by each culture condition. In addition, the media optimization experiments were performed with sterile soil, which may not accurately reflect the microbial competition present in natural environments. Future studies should utilize non-sterile soils during media optimization to better reflect natural ecological conditions.”

12. Line 488: yes, please discuss why this wasn’t also considered in the comparisons of ASH and Acer agars

We thank the reviewer for this valuable comment. In our study, we directly compared Ashdown agar and ACER agar using environmental samples spiked with 10 distinct B. pseudomallei strains. The results indicated that the colony counts obtained on both media were not significantly different, suggesting comparable sensitivity in terms of bacterial recovery. However, colony morphology on Ashdown agar was generally easier to recognize and distinguish. In addition, the practical aspects of media preparation favored Ashdown agar. Preparation of Ashdown agar is relatively straightforward, whereas ACER agar requires multiple additional components including buffer, microsalt solution, MEM vitamins, and antibiotics that making it more complex and resource-intensive to prepare.

In Results Lines 297-299: “The recovery of *B. pseudomallei* from PBS was assessed by culturing ten environmental strains and the clinical strain K96243 on Ashdown and ACER agar in three independent experiments.”

In Discussion Lines 499-509: “Comparison of the growth of *B. pseudomallei* on Ashdown and ACER agar showed that Ashdown agar performed better than ACER agar in terms of growth and provided a more distinct colony morphology of *B. pseudomallei*. These findings are consistent with previous reports, which emphasized using Ashdown agar for presumptive identification, based on the distinct wrinkled and purple colony appearance characteristic of *B. pseudomallei* (Chantratita, Wuthiekanun et al. 2007). This particular colony appearance is attributed to components of Ashdown agar, including crystal violet, which contributes to the purple coloration of *B. pseudomallei* colonies, and neutral red, which enhances their distinctive wrinkled appearance (Ashdown 1979). Although ACER agar supported comparable bacterial counts, differentiation was challenging due to the less distinct colony form, necessitating confirmation tests.”

13. Line 501: is there any evidence of how long IgG detected in assay lingers? Where the results published in UR from a study that was also conducted in the dry season? Perhaps this community, as the soil prevalence also perhaps suggest, just less endemic that UR?

We appreciate the reviewer’s comment. The previous studies of IgG responses in melioidosis are limited. However, Pumpuang et al. (2019) conducted a longitudinal study of melioidosis patients from day 0 to day 28 after enrolment, which demonstrated that Hcp1-specific IgG remained at high levels throughout this period. These findings suggest that IgG can persist for at least two months following infection.

In Trung et al. (2011) and Wuthiekanun et al. (1995), the studies conducted in Ubon Ratchathani were carried out during the dry season; however, they were not conducted in the exact same months as our study.

We agree that our comparatively lower detection rates may also reflect a genuinely lower level of endemicity in Amnat Charoen compared with Ubon Ratchathani. This interpretation is consistent with both the lower seroprevalence observed in our study population and the reduced environmental isolation rates we recorded. We have clarified this point in the Discussion (Lines 533-538, 629-633)

Lines 533-538: “Our study revealed an overall seropositivity rate of 8.5% among seropositive individuals that included individuals with probable exposure to *B. pseudomallei* (7.0%) and individuals with probable melioidosis (1.5%).This finding contrasts with a previous study conducted in Ubon Ratchathani, which reported a high seroprevalence rate of 38% among healthy individuals, based on indirect hemagglutination assay (IHA) at a cutoff titer of 1:80 (Chaichana, Jenjaroen et al. 2018).”

Lines 629-633: “Finally, at some sampling locations and during certain periods, the soil was compacted or difficult to dig, which posed challenges for sample collection and may have limited the number of samples obtained. Therefore, sampling should ideally be conducted during the monsoon season, when soil conditions are more favorable.”

14. Lines 555-561: explain why PCR wasn’t conducted on pre-enriched or culture samples given authors have cited literature that reports better sensitivity

We thank the reviewer for this comment. In our study, PCR was performed directly on soil samples. The rationale was that if real-time PCR could detect positive soil samples directly, it would reduce time and labor by bypassing several lengthy steps. Specifically, pre-enrichment requires 5–9 days in ACER broth, or other selective media also require 2-3 days, while culture alone requires approximately 3–4 days. Using direct soil therefore offered the potential advantage of faster detection compared to pre-enriched or cultured samples. However, our results demonstrated low sensitivity, which we have acknowledged in the Discussion (Lines 627-629) as a limitation of the study.

Lines 627-629: “Future studies should consider using prior enrichment or soil pellet for DNA extraction and incorporate validated internal controls to enhance the accuracy and reliability of real-time PCR assays.”
